# Simultaneous Estimation of Gas Adsorption Equilibria and Kinetics of Individual Shaped Adsorbents

**DOI:** 10.1021/acs.chemmater.2c01567

**Published:** 2022-07-27

**Authors:** Hassan Azzan, Ashwin Kumar Rajagopalan, Anouk L’Hermitte, Ronny Pini, Camille Petit

**Affiliations:** † Department of Chemical Engineering, Imperial College London, London SW7 2AZ, United Kingdom; ¶ Department of Materials, Imperial College London, London SW7 2AZ, United Kingdom

## Abstract

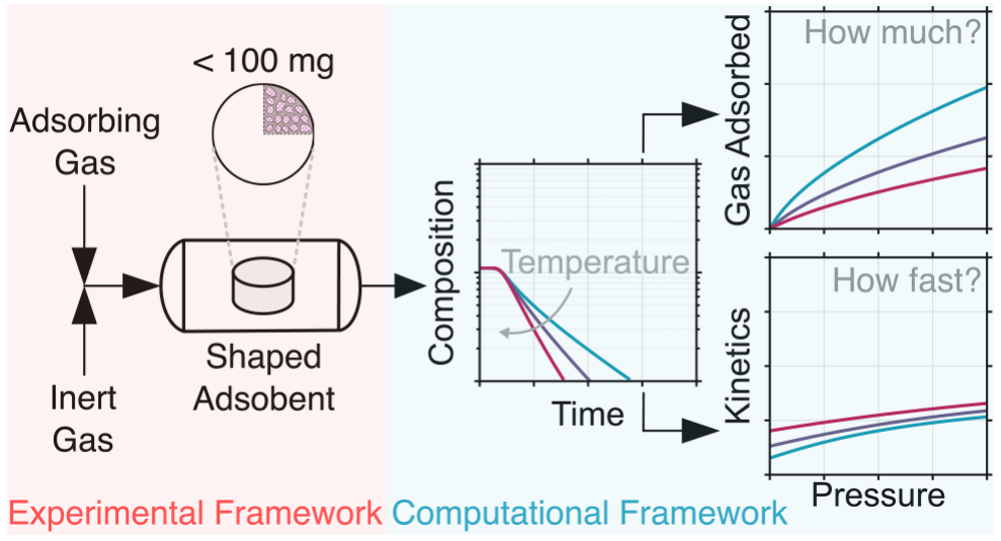

Shaped adsorbents (e.g., pellets, extrudates) are typically employed in several gas separation and sensing applications. The performance of these adsorbents is dictated by two key factors, their adsorption equilibrium capacity and kinetics. Often, adsorption equilibrium and textural properties are reported for materials. Adsorption kinetics are seldom presented due to the challenges associated with measuring them. The overarching goal of this work is to develop an approach to characterize the adsorption properties of individual shaped adsorbents with less than 100 mg of material. To this aim, we have developed an experimental dynamic sorption setup and complemented it with mathematical models, to describe the mass transport in the system. We embed these models into a derivative-free optimizer to predict model parameters for adsorption equilibrium and kinetics. We evaluate and independently validate the performance of our approach on three adsorbents that exhibit differences in their chemistry, synthesis, formulation, and textural properties. Further, we test the robustness of our mathematical framework using a digital twin. We show that the framework can rapidly (i.e., in a few hours) and quantitatively characterize adsorption properties at a milligram scale, making it suitable for the screening of novel porous materials.

## 1 Introduction

The application of adsorption on porous materials has been studied for a variety of uses such as gas separation, storage, and sensing.^1−3^ The prediction of the performance of these porous materials in these applications is typically carried out using optimization of computational models that simulate the respective processes that can then be validated with experiments.^4−13^ The reliability of the results obtained from these simulations is contingent on the accurate determination of the textural characteristics, e.g., skeletal density and porosity, and of the adsorption behavior, i.e., equilibrium adsorption capacity and kinetics for the relevant gases on the porous material. Recent advances in material science and computational methods has led to an increase in the rate of material discovery for various applications.^14−17^ Reticular chemistry, e.g., for metal–organic frameworks (MOFs), enables modifying the structure of a given material by adding, mixing, and/or matching different functional groups, which leads to a large number of discovered or potentially discoverable porous materials. Typically, these new materials are synthesized in small lab-scale quantities, i.e., on the order of 1 to 2 g. The issues surrounding the small quantity of material synthesized and the ever expanding studies that report the discovery of novel porous materials makes the experimental characterization of textural characteristics and adsorption behavior a challenging and laborious task. However, for novel materials, to design better formulations for shaped adsorbents (e.g., pellets, granules, extrudates, monoliths), it is necessary to obtain and report these characteristics. Nevertheless, these characteristic properties are generally not characterized to a sufficient extent when reporting new adsorbent materials. Therefore, the need of the hour is to develop and validate rapid techniques that encompass all these characteristics to provide a thorough characterization of the shaped adsorbents.

The textural characterization of newly synthesized porous materials is commonly carried out by either subatmospheric argon or nitrogen adsorption physisorption experiments at 87 K or 77K, respectively.^18^ This analysis is limited to a pore diameter *D* ≤ 50 nm and cannot resolve accurately larger pores generated upon shaping the material into pellets, granules, etc. (up to a pore diameter of 400 μm). These large pores can be characterized by mercury intrusion porosimetry (MIP).^19^ The combination of physisorption methods with MIP can thus elucidate the pore characteristics of shaped adsorbents, providing a practical means to obtain the total porosity and the skeletal density of a few milligrams of the given porous material.^20^ Note that, in the field of adsorption, it is common practice to estimate the skeletal density of the material using helium adsorption (or helium pycnometry). However, it is known that this particular method leads to inaccurate and inconsistent estimates for the skeletal density of microporous materials.^20−22^

The characterization of adsorption behavior falls under two categories, i.e., adsorption equilibrium and adsorption kinetics. Typically adsorption equilibria, specifically for a single component, is measured using volumetric and/or gravimetric techniques.^23−25^ Techniques to measure both adsorption equilibria and kinetics can be broadly classified into three approaches. The first approach, grouped under “batch” techniques, involves using purpose-built or commercial static adsorption instruments as is or with modifications to the instrument with a few milligrams of the sample. These can include both volumetric and gravimetric techniques, which require tracking the uptake of adsorbate over time in a closed system.^26−29^ These techniques are often slow, even for pure component equilibrium measurements (up to 2–3 days to obtain a single isotherm), making them unsuitable for rapid adsorbent characterization. The second approach, referred to as a dynamic column breakthrough (DCB) technique, involves performing dynamic gas sorption experiments in a purpose-built adsorption column with a few grams to kilograms of the sample. Despite the large quantity of sample required, these setups enable studying competitive adsorption, adsorption kinetics, and complex mass, momentum, and heat transfer dynamics in a packed bed column.^7,30−35^ The third approach, referred to as zero length column (ZLC) technique, involves performing dynamic gas sorption experiments in an purpose-built adsorption column with a few milligrams of the sample.^36−38^ This technique facilitates a reliable extraction of information on adsorption equilibrium and diffusion behavior, particularly for strongly adsorbing gases.^37^ The small quantity of material used gives the advantage of being able to assume isothermal operation of the system and neglect effects related to axial variations of composition, temperature, and pressure. The ZLC technique has been widely adopted ever since it was proposed, and it has been applied to study adsorption of liquids and gases on powders, pellets, and other structured forms.^37^ Recently, simultaneous estimation of equilibrium and kinetics by fitting time-resolved ZLC responses to a fully descriptive mathematical model has been proposed.^39,40^ All of the aforementioned techniques typically require fitting time-resolved responses from experiments carried out at different conditions to a mathematical model that describes the physics of the system in order to extract thermodynamic and kinetic properties of the system. Irrespective of the technique, the discrete experimental data is always converted to a functional form using thermodynamically consistent or empirical isotherm and kinetic models to facilitate incorporation of adsorption characteristics of a given material into process or sensor models.^6,13^

Over the past few years, there has been a strong momentum toward developing robust and practical approaches to rapidly characterize the adsorption behavior of gases in porous materials. The overarching goal of this work is to propose a technique—drawing inspiration from the DCB and the ZLC techniques—to address the challenge related to rapid and accurate characterization of both adsorption equilibrium and kinetics using small amounts of sample (<100 mg). A technique like the one proposed in this work will be ideally suited for large-scale material screening. The approach proposed in this work addresses the challenges commonly encountered with the aforementioned techniques, associated with the time taken to characterize the material properties and with the quantity of sample required for a thorough characterization. Most importantly, we provide a general engineering framework for material characterization, instead of developing a tailor-made experimental and mathematical approach, thereby guaranteeing easy transferability and reproducibility for future practitioners.

To achieve this goal, we have developed a combined experimental and modeling framework for individual shaped adsorbents. The accurate characterization of the textural properties, i.e., the skeletal density and porosity, of the shaped adsorbents is a key prerequisite to quantify the adsorption behavior using the approach presented in this work. To quantify the textural properties, we have used a combination of N_2_ sorption and MIP measurements (see Section 2.2.1). To quantify the adsorption behavior, we have developed a dynamic sorption experimental setup, and we have complemented it with detailed mathematical models (see Section 3). The quantitative performance of the experimental and modeling framework has been evaluated by characterizing the shaped form (in this work in the form of pellets) of three different materials that exhibit differences in their chemistry, synthesis, formulation, and textural properties (see Section 2.1). We have compared the adsorption equilibrium of CO_2_ obtained from a commercial volumetric setup (see Section 2.2.2) with the one obtained from the dynamic sorption experimental setup proposed in this work. Additionally, we have tested the robustness of the simulation framework developed in this work and the sensitivity of model inputs using a digital twin (see Section 5). The unified characterization pipeline enables experimental characterization of adsorption behavior on adsorbents using milligram scale samples over a wide range of pressures and temperatures on the order of a few hours (see Section 4.2).

## 2 Materials and Material Characterization

### 2.1 Materials

We used activated carbon Norit RB3 rods (AC) as supplied by a manufacturer (Sigma-Aldrich, Germany) for the experiments presented in subsequent sections. The rods are cylindrical with an approximate diameter of 1 mm and a height of 3 mm. We synthesized boron nitride (BN) in its powder form, using a methodology previously reported.^41^ Further, we obtained the shaped form of BN, by pelletizing the synthesized powder. To achieve this, we placed 50 mg of BN powder, without any binder, into a 5 mm pellet die, which was then placed in a Specac Manual Hydraulic Press (Specac Limited, U.K.). We applied a load of 0.4 t on the die and maintained it for 30 s to form cylindrical pellets with a diameter of 5 mm and an approximate height of 4 mm. We obtained the powder form of molecular sieve 13X (13X) powder from a manufacturer (Sigma-Aldrich, Germany) and subsequently pelletized it to obtain its shaped form. To achieve this, we followed the same pelletization procedure as that of BN, but using 80 mg of 13X powder, without any binder to form pellets of a diameter of 5 mm and an approximate height of 3 mm. Prior to performing the experiments reported in the subsequent sections, we degassed all the three materials using the protocol described in Section S2.2 in the Supporting Information.

### 2.2 Material Characterization

In this section, we present the techniques and the corresponding analysis methodology used to characterize the textural properties, i.e., skeletal density and crystallinity (Section 2.2.1), and adsorption equilibria of CO_2_ using a commercial volumetric setup (Section 2.2.2), on the three adsorbents evaluated in this work.

#### 2.2.1 Textural Properties

To obtain the porosity and skeletal density of the adsorbents, we used a combination of N_2_ sorption measurements at 77 K and MIP. We performed the N_2_ sorption measurement using Autosorb iQ (Quantachrome Instruments, U.S.A.) in the pressure range of 3 × 10^–7^ bar to 1 bar. Subsequently, we obtained the pore size distribution (PSD) for the materials using the adsorption branch of the N_2_ isotherm with the nonlocal density functional theory (NLDFT) model. We used a cylindrical/spherical pores on carbons model for AC, slit pores on carbons model for BN, and cylindrical/spherical pores on silica/zeolite model for 13X. We used the proprietary data analysis software (ASiQwin version 5.2, Quantachrome Instruments, U.S.A.) supplied by the manufacturer to fit the experimental data with the corresponding model for all three materials. Note that we obtained a fitting error below 2% for all three materials. Finally, we converted the PSD to a cumulative pore volume as a function of the pore diameter for further analysis (see Section 4.1). We performed the MIP measurements on all three materials using an AutoPore IV Series Mercury Porosimeter (Micromeritics Instrument Corporation, U.S.A.) in the pressure range of 3.7 × 10^–2^ bar to 2.23 × 10^3^ bar. Subsequently, we obtained a cumulative mercury intrusion volume, which can be translated to a cumulative pore volume for further analysis, as a function of the pore diameter using the proprietary measurement and analysis software AutoPore IV (version 9500) supplied by the manufacturer. Prior to commencing both the measurements, we performed an *ex situ* degassing of all the samples using the protocol outlined in Section S2.2 in the Supporting Information, corresponding to a given material.

**Figure 1:**
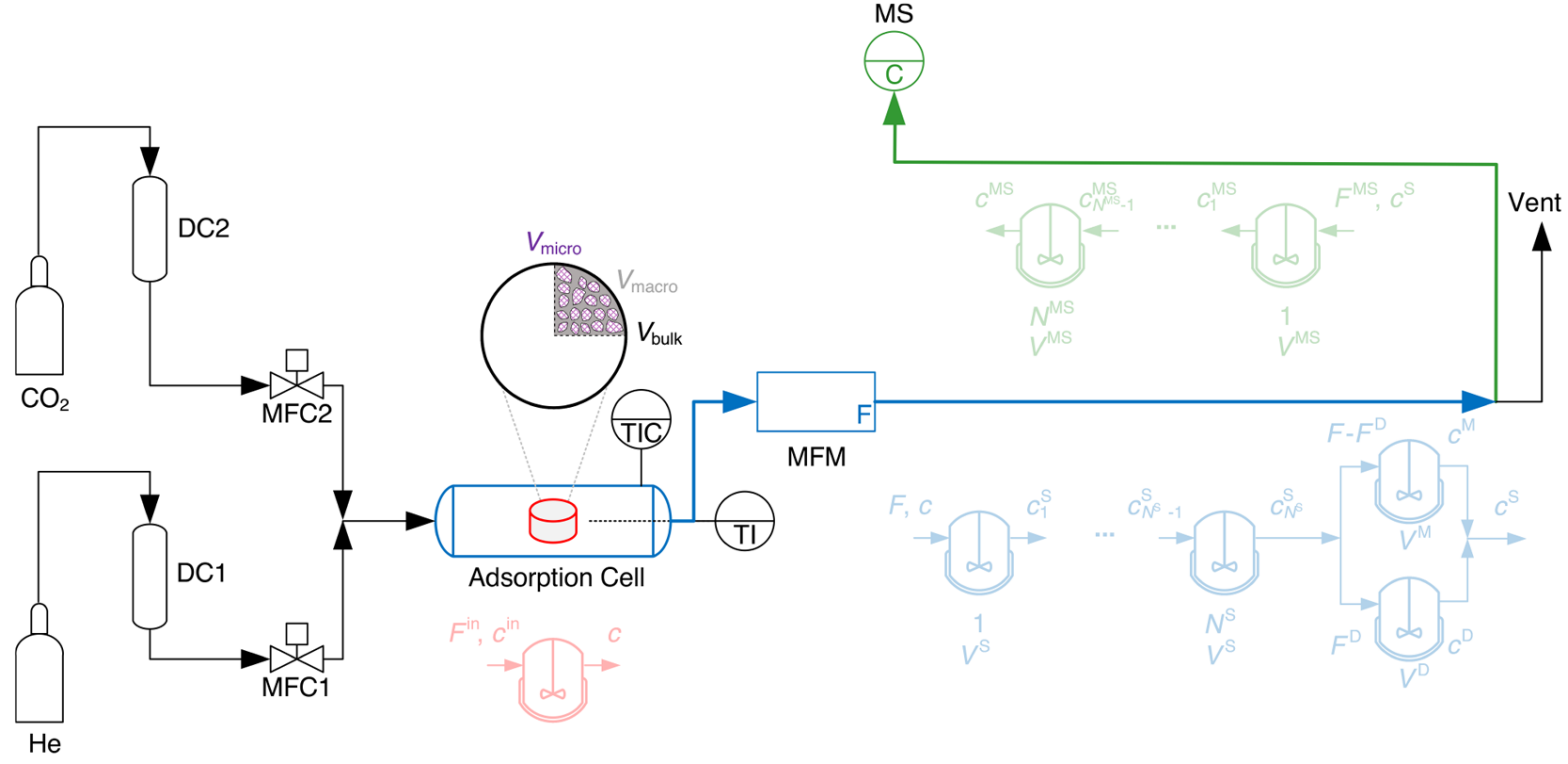
Schematic of the experimental setup used to simultaneously estimate the equilibrium and kinetics of porous materials. Gas at different compositions is prepared by varying the flow rates in the mass flow controllers (MFC). A porous material sample in a pellet form is placed in the adsorption cell (red). The temperature of the adsorption cell is monitored (TI) and controlled (TIC) using a heating tape. The evolution of the gas flow rate and composition is monitored using a mass flow meter (MFM, blue) and mass spectrometer (MS, green), respectively. The entire setup is divided into three distinct segments for modeling purposes, described in Section 3.2, i.e., the adsorbent pellet (red), adsorption cell, MFM, and the gas lines (blue), and the MS (green). These segments are modeled as continuous stirred tank reactors (CSTRs), and they are visualized in a lighter shade underneath each section with their corresponding volumes, flow rates, and gas inlet/outlet concentrations.

Using these measurements, we computed the skeletal density ρ_s_ [g cm^–3^] and the corresponding total porosity or voidage ϵ_T_ [−] of the material as follows
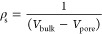
1a
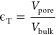
1bwhere *V*_pore_ [cm^3^ g^–1^] is the total pore volume for each material (sum of the micropore volume *V*_micro_, mesopore volume, and macropore volume *V*_macro_ as shown in Figure 1) and *V*_bulk_ [cm^3^ g^–1^] (shown as a black envelope in Figure 1) is the bulk volume. The former is obtained by combining the cumulative pore volume as a function of the pore diameter from the two measurements, and the latter is obtained by using the MIP measurement at 0.37 kPa.

Additionally, we characterized the crystallinity of the materials using X-ray diffraction (XRD). We measured the XRD patterns on the shaped forms using a PANalytical X’Pert PRO diffractometer (Malvern Panalytical Ltd., U.K.).

#### 2.2.2 Volumetric Adsorption Equilibrium

To validate the accuracy of the adsorption equilibrium data obtained using the dynamic sorption experiments (see Section 4.2), we obtained independent adsorption equilibrium measurements of CO_2_ on the three materials. To this aim, we used the same commercial volumetric setup used to obtain the N_2_ sorption data. We performed measurements in a pressure range of 1 × 10^–3^ bar to 1.013 bar at three different temperatures, specifically at 293, 303, and 313 K. Before performing the measurements, we degassed all the materials following the protocol outlined in Section S2.2 in the Supporting Information. We subsequently fitted the experimental data to standard adsorption equilibrium models reported in the literature. For AC and 13X, we used a dual-site Langmuir (DSL) model, and for BN, we used a single-site Langmuir (SSL) model. Note that the SSL model is a subcase of the DSL model, as will be explained below. The DSL model used in this work is given as follows
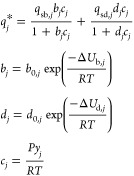
2where *q*_j_^*^ [mol kg^–1^] is the absolute amount of gas *j* adsorbed on the adsorbent, *c*_*j*_ [mol m^–3^] is the bulk phase gas concentration calculated using the ideal gas law at a given gas mole fraction *y*_*j*_ [−], an absolute pressure *P* [Pa], and an absolute temperature *T* [K], *R* [J mol^–1^ K^–1^] is the universal gas constant, *q*_sb,*j*_ [mol kg^–1^] and *q*_sd,*j*_ [mol kg^–1^] are the saturation capacities for the two energetically heterogeneous adsorption sites, and *b*_*j*_ [m^3^ mol^–1^] and *d*_*j*_ [m^3^ mol^–1^] are the temperature dependent adsorption coefficients for the corresponding sites, each described by an Arrhenius expression with two constants, *b*_0,*j*_ [m^3^ mol^–1^] and −Δ*U*_b,*j*_ [J mol^–1^] for the first site and *d*_0,*j*_ [m^3^ mol^–1^] and −Δ*U*_d,*j*_ [J mol^–1^] for the second site, respectively. When we fit the experimental data using the SSL model, we neglect the second site in the DSL model, i.e., *q*_sd,*j*_ = 0, *d*_0,*j*_ = 0, and −Δ*U*_d,*j*_ = 0, thereby reducing the DSL model to a SSL model. Note that since we are measuring the experimental data at subatmospheric and atmospheric conditions, we do not make a distinction between excess and absolute adsorption.

To estimate the isotherm parameters, we used a parameter estimation routine. First, we preprocessed the raw experimental data using the steps outlined in Section S3 in the Supporting Information. Second, we fitted the preprocessed experimental equilibrium data to either the SSL or the DSL model using a maximum likelihood estimator (MLE), described in Section S4 in the Supporting Information. In eq S2 in the Supporting Information, for this particular case, *ŷ*_*k*_ is defined as the *k*th point of the experimentally measured absolute amount of gas adsorbed *q̂*_*j*,k_^*^ for gas *j* and *y*_*k*_(**θ**) is defined as the corresponding model prediction *q*_*j*,k_^*^(**θ**) for a given isotherm parameter vector **θ**. For the SSL model, the isotherm parameter vector **θ** = [*q*_sb,*j*_*b*_0,*j*_ −Δ*U*_b,*j*_], and for the DSL model, the isotherm parameter vector **θ** = [*q*_sb,*j*_*b*_0,*j*_ −Δ*U*_b,*j*_*q*_sd,*j*_*d*_0,*j*_ −Δ*U*_d,*j*_]. We obtained the local minimizer θ* by minimizing the objective function *J*(θ), given by eq S2 in the Supporting Information. We solved this minimization problem using a global optimization routine (MATLAB’s globalsearch) coupled with a local sequential quadratic programming routine (MATLAB’s fmincon) in MATLAB R2020a (The Mathworks Inc., U.S.A.). At the local minimizer, we also computed the confidence intervals for each parameter in the model at a given confidence level η using eq S3 in the Supporting Information.

## 3 Simultaneous Estimation of Adsorption Equilibrium and Kinetics

The key aim of this work is to develop a robust experimental and computational framework to simultaneously predict adsorption equilibria and kinetics using a small amount of shaped adsorbent material. To address this, we have built a dynamic gas sorption experimental setup taking inspiration from two methods, namely, zero length chromatography (ZLC)^37^ and dynamic column breakthrough (DCB).^33^ We provide a detailed discussion on the similarities and the dissimilarities between the proposed dynamic gas sorption setup and those reported in the literature, e.g., ZLC and DCB, in Section 5.

As in the case of ZLC and DCB, to obtain equilibrium and kinetic parameters, we perform dynamic gas sorption experiments at different flow conditions and initial gas compositions. A typical experiment is performed as follows: (1) saturate the adsorbent with the adsorbing gas of interest at a given partial pressure and temperature; (2) switch to a pure inert gas and track the desorption profile of the adsorbing gas using a gas detector; and (3) repeat the aforementioned steps at different saturation partial pressures, inert gas flow rates, and temperatures.

Upon performing the experiments, we used the time-resolved experimental responses and performed a full curve fit to obtain adsorption equilibrium and kinetic parameters using a parameter estimator that is wrapped around a detailed mathematical model. The experimental framework used to perform these experiments is described in detail in Section 3.1. The mathematical framework used to explain the mass transport in the system is described in Section 3.2. Finally, the simulation framework to obtain the adsorption equilibrium and kinetic parameters is described in detail in Section 3.3. A visual schematic illustrating this experimental framework along with its mathematical equivalent is shown in Figure 1.

### 3.1 Experimental Framework

#### 3.1.1 Design of Experimental Setup

We divided our experimental setup into three distinct sections: (1) Gas supply system, (2) Segment I, shown in blue in Figure 1, and (3) Segment II, shown in green in Figure 1. *Segment I* is composed of the adsorption cell, the mass flow meter (MFM), and the gas lines to the point the stream is split into two (for the mass spectrometer (MS) and the vent). *Segment II* is composed of the MS and the gas line from the stream split to the MS. In this work, *Segment I* and *Segment II* will be referred to as the blank volume, and the rationale behind this choice is explained in detail in Section S5.2 in the Supporting Information. Photographs of the setup are shown in Figure S2 in the Supporting Information.

##### 3.1.1.1 Gas Supply System

We designed the gas supply system for the setup as follows. The experimental setup is connected to two high pressure gas cylinders, namely, CO_2_ (the adsorbing gas, >99.995%, BOC, United Kingdom) and He (the inert gas, >99.999%, BOC, United Kingdom). The outlets from the regulated gas cylinders are passed through purpose built drying columns (DCs) to remove any moisture present in the gas. Further details about the construction of the drying columns are provided in Section S2.3 in the Supporting Information. The gas flow rates of the two gases are controlled using two mass flow controllers (MFC), one for CO_2_ (Alicat MC-200SCCM-D, Alicat, U.S.A.), and one for He (Alicat MC-500SCCM-D, Alicat, U.S.A.). The calibration standards for the corresponding gas, provided by the manufacturer, were used to accurately regulate the mass flow rates. Note that a pressure of 1 bar is maintained at the outlet of both the MFCs. CO_2_ and He were mixed in a tee connector (PEEK Low Pressure Tee Assembly 1/16”, IDEX Health & Science LLC, U.S.A.), at a predetermined mass flow, regulated by the two MFCs. The switch between the mixture and the pure inert gas is performed by setting the CO_2_ mass flow rate to zero, which in our system was found to be instantaneous. An elegant alternative to this would be to incorporate a six-way valve as has been used in other studies.^42,43^ For the case of the blank experiments, where the gas is switched between two pure gas streams, a switching valve is necessary. Hence, to ensure a near instantaneous switch for a step change in gas composition, a 6-port 2-position switching valve (VICI Valco, U.S.A.) was used.

##### 3.1.1.2 Segment I

The tee junction is connected to the adsorption cell via PEEK Tubing (OD 1/16” and ID 0.04”, IDEX Health & Science LLC, U.S.A.). This choice of tubing was made to minimize the volume upstream of the adsorption cell. The adsorption cell was constructed using widely available parts. It consists of a stainless steel 1/16” to 1/8” reducing union (Hy-Lok, United Kingdom) followed by a 1/4” Swagelok F series stainless inline filter (Swagelok, U.K.) that is modified, by removing the replaceable internal elements, to hold the adsorbent material within. A stainless steel wire mesh disc was placed at the outlet end of the filter to prevent any particulates from being carried downstream. Additionally, a stainless steel ball bearing is placed inside the filter along with the adsorbent material to allow rapid heat dissipation and to thereby maintain isothermal operation.^27^ The outlet from the adsorption cell is then connected to a stainless steel tee connector through which an inline K-type thermocouple (Model SCASS-062G-12, Omega, U.K.) is inserted. This thermocouple is positioned downstream of the aforementioned protective wire mesh. The internal temperature of the adsorption cell was logged using a data logger (TC-08, Pico Technology, U.K.) via the propriety software Picolog 6 (version 6.1.18, Pico Technology, U.K.). The mean of this recorded temperature for each experiment is used as the temperature input to the models described in Section 3.2. Isothermal operation at above-ambient temperatures is achieved via external heating of the adsorption cell using a fiberglass heater tape (Model Number SWH171-020, Omega, U.K.). A PID heater controller using a second K-type thermocouple attached to the exterior of the adsorption cell beneath the heating element is used to ensure good control of the temperature. The remaining outlet from the tee connector is connected to a mass flow meter (MFM, Alicat M-1 SLPM-D, Alicat, U.S.A.). A recalibration of the MFM was performed for mixtures of He and CO_2_ to obtain mass flow rates of mixtures used in the experiments, which is described in detail in Section S2.5 in the Supporting Information.

##### 3.1.1.3 Segment II

The gas stream exiting the MFM is split into two streams, one to a vent and another to a stainless steel capillary tube leading to a mass spectrometer (MS, OmniStar Gas Analysis Model GSD 320 O1, Pfeiffer Vacuum, Switzerland). The gas is sampled in real-time, throughout the course of the experiment, at a constant flow rate *F*^MS^ = 0.4 cm^3^ min^–1^. The resulting ion current data is logged for the two gases from the MS using the manufacturer supplied propriety software (QUADERA Version 4.62, Pfeiffer Vacuum, Switzerland). A calibration of the MS was performed for mixtures of He and CO_2_ to allow the conversion of the raw ion signal to gas phase composition, as discussed in Section S2.4 in the Supporting Information.

To provide set points for the MFCs and log the flow rates from the MFCs and MFM (both their calibration and experimental runs), the temperature, and the MS signal, a desktop computer running MATLAB R2020a was used (The Mathworks Inc., U.S.A.). The entire setup was automated and the data analysis was performed using an in-house software package, developed in MATLAB R2020a and Python 3.8.5, which is accessible through a dedicated Github repository.

#### 3.1.2 Experimental Procedure

##### 3.1.2.1 Gas Adsorption in the Adsorbent

To estimate the adsorption equilibria and kinetics on the different adsorbents, we performed dynamic gas sorption experiments using the setup described in Section 3.1.1. In the first step of the dynamic sorption experiments, we saturated the adsorbents at a given partial pressure of the gas and temperature. In the second step, we switched to a pure inert gas at a given flow rate and tracked the desorption profile. In this work, we used two different inert gas (helium) flow rates *F*^in^ = [10.0 60.0]^T^ cm^3^ min^–1^. At *F*^in^ = 10.0 cm^3^ min^–1^, we saturated the adsorbents with CO_2_ at a partial pressure *p* = *Py*_0_ = [0.12 0.94]^T^ bar. At *F*^in^ = 60.0 cm^3^ min^–1^, we saturated the adsorbents at a partial pressure *p* = *Py*_0_ = [0.11 0.73]^T^ bar. We performed these experiments at a total pressure *P* = 1 bar and at three different temperatures *T* = [306 325 345]^T^ K. In this work, we chose these three values as they cover the range of temperatures typically encountered under process conditions (i.e., feed temperature and temperature swings in a packed column). Note that the methodology proposed in this work is not limited to the aforementioned three temperatures.

##### 3.1.2.2 Blank Volume

The characterization of the blank volume in a dynamic sorption experimental setup is a key prerequisite for the accurate estimation of the adsorption behavior in the adsorbents. The procedure for these experiments is detailed in Section S5.1 in the Supporting Information.

In all the experiments performed in this work, we tracked the evolution of the CO_2_ gas composition during its desorption from the adsorbent using the MS.

### 3.2 Mathematical Framework

#### 3.2.1 Gas Adsorption in the Adsorbent

Here, we formulate a mathematical model for gas adsorption in the adsorbent shown in Figure 1 (red). Following the zero length column approximation,^37^ we have assumed the adsorbent to be a continuous stirred tank reactor (CSTR). This approximation holds true when the length of a packed bed column in an adsorption/chromatographic system tends to zero. Additionally, we formulate the equations at an isobaric and isothermal condition. Under these assumptions, the component mass balance for the gas *j* is written as
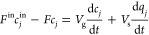
3where *F*^in^ [m^3^ s^–1^] and *F* [m^3^ s^–1^] are the volumetric mixture gas flow rate at the inlet and the outlet of the CSTR, respectively, *c*_*j*_^in^ [mol m^–3^] and *c*_*j*_ [mol m^–3^] are the concentration of the gas *j* at inlet and outlet of the reactor cell, respectively, *q*_*j*_ is the amount of gas adsorbed in the adsorbent at time *t*, *V*_g_ [m^3^] is the gas phase volume, and *V*_s_ [m^3^] is the skeletal volume of the adsorbent. The latter two quantities are computed as
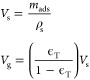
4where *m*_ads_ [kg] is the mass of the adsorbent, ρ_s_ is the skeletal density of the adsorbent, obtained from eq 1a, and ϵ_T_ is the total porosity, obtained from eq 1b.

We assume the rate of uptake of gas *j* in the adsorbent to be described using the linear driving force (LDF) model as
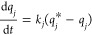
5where *q*_j_^*^ is the equilibrium adsorption capacity, obtained from eq 2, and *k*_*j*_ [s^–1^] is the lumped kinetic rate constant for a given gas *j* that describes the resistance to mass transfer from the gas phase to the solid phase.

We have incorporated two contributions that are analogous to a micropore and a macropore equivalent resistance in the lumped kinetic rate constant *k*_*j*_. This lumped contribution has a mathematical structure similar to the Glueckauf approximation,^44^ often used in adsorption process modeling, albeit without explicitly accounting for the radius of the crystals in the case of micropore resistance and radius of the pellet, porosity, molecular diffusivity, and tortuosity in the case of macropore resistance. Nevertheless, not accounting explicitly for these quantities should not influence the outcome of this work, as in most studies reported in the literature these are assumed to be a constant. This lumped kinetic rate constant *k*_*j*_ is given as
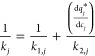
6where *k*_1,*j*_ [s^–1^] and *k*_2,*j*_ [s^–1^] are lumped rate constants analogous to micropore and macropore resistances and  [−] is the local slope of the isotherm at concentration *c*_*j*_.

Finally, to close the system of equations, we impose a mass conservation constraint using
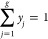
7

When we have a binary mixture of gases, one adsorbing and the other inert, at time *t* = 0, the adsorbent is assumed to be saturated at a given pressure *P* [Pa], temperature *T* [K], and adsorbing gas mole fraction *y*_*j*_^0^ [−]. Additionally, under the assumption of negligible pressure drop, using the equation of state given in eq 2, we can reformulate eqs 3–7 to describe a desorption process using an inert gas as follows
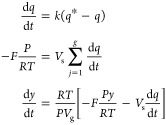
8where these equations are written for the adsorbing gas with the following initial conditions
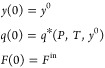
9

#### 3.2.2 Blank Volume

The resulting response from the adsorbent is propagated through a blank volume model that accounts for the extra volumes in the setup. This model is described in detail in Section S5.1 in the Supporting Information.

### 3.3 Simulation Framework

#### 3.3.1 Solution of the Model

We employ the following steps in a sequential manner to simulate a desorption experiment performed using the setup shown in Figure 1:

1. We describe the desorption of a strongly adsorbing gas in the adsorbent using eq 8, when it is subjected to a step concentration to an inert gas, to obtain a *true* response.
2. We subject the *true* response from point 1 through the blank volume model of the setup, composed of two distinct segments. These segments add a delay and dispersion to the *true* response. Here,
  (a) we propagate the true response through *Segment I* of the blank volume, modeled using eqs S4–S6 in the Supporting Information.
  (b) we propagate the response from (a) through *Segment II* of the blank volume, modeled using eq S8 in the Supporting Information, to obtain the final *composite* response.

Note that, for simulating a blank experiment, we directly go to point 2, in which the *true* response at the inlet of *Segment I* corresponds to a step change in the gas composition, due to the absence of an adsorbent within the adsorption cell.

To integrate the model equations that describe gas adsorption in the adsorbent, given by eq 8, and to integrate the model equations that describe the blank volume in the system, given by eqs S4 and S5 in the Supporting Information (*Segment I*) and eq S8 in the Supporting Information (*Segment II*), we use a stiff solver in the *solve*_*ivp* function of the scipy package in Python 3.8.5.^45^ for a time span *t*_int_ [s].

#### 3.3.2 Parameter Estimation

We developed a parameter estimation methodology to estimate the model parameters to describe the blank volume and the adsorption behavior in the adsorbents. We obtained the blank volume model parameters using the approach presented in Section S5.3 in the Supporting Information. Upon estimating these parameters, we obtained the adsorption equilibria and kinetic model parameters by simulating the combined models for the gas adsorption in the adsorbent and the blank volume. Prior to performing parameter estimation, we preprocessed the raw experimental data, obtained using the protocol described in Section 3.1.2, through the steps outlined in Section S3 in the Supporting Information. We obtained the minimizer θ*, using a maximum likelihood estimator described in Section S4 in the Supporting Information, for the model corresponding to the adsorption behavior in the adsorbents. In eq S2 in the Supporting Information, *ŷ*_*k*_ is defined as the *k*th point of the experimentally measured outlet CO_2_ gas composition obtained from the MS and *y*_*k*_^MS^(**θ**) is defined as the corresponding model prediction for a given model parameter vector **θ**. When using the SSL model to describe equilibrium, the model parameter vector **θ** = [*q*_sb,*j*_*b*_0,*j*_ −Δ*U*_b,*j*_*k*_1,*j*_*k*_2,*j*_], and when using the DSL model to describe equilibrium, the model parameter vector **θ** = [*q*_sb,*j*_**b*_0,*j*_ −Δ*U*_b,*j*_*q*_sd,*j*_*d*_0,*j*_ −Δ*U*_*d*,*j*_k*_1,*j*_*k*_2,*j*_]. We obtained the local minimizer θ* by minimizing the objective function *J*(θ), given by eq S2 in the Supporting Information. We solved this minimization problem using an evolutionary optimization algorithm, i.e., a standard and elitist genetic algorithm (geneticalgorithm2^46^ (v6.2.4) in Python 3.8.5). At the local minimizer, we also computed the confidence intervals for each parameter in the model at a given confidence level η using eq S3 in the Supporting Information.

We acknowledge that obtaining the true minimizer of the model might not be guaranteed by the chosen optimization algorithm. We can attribute this to factors like structure of the problem and genetic algorithm parameter values (crossover, mutation, etc.), to name a few. Therefore, to ensure we move toward the true minimizer, we have structured each parameter estimation run to consist of five local iterations. We executed each of these local iterations for a total of 15 (for adsorption behavior) generations with a population size of 400 (for adsorption behavior). The initial population used for each of the local iterations comes from the final population of the previous local iteration (except for the first iteration). The model parameter vector at the end of the fifth iteration is assumed to be the minimizer θ*. The upper and lower bounds used for each of the parameters, along with their corresponding type (discrete or continuous), is provided in Table S2 in the Supporting Information.

Finally, when estimating the model parameters to describe the adsorption behavior in the adsorbents, we additionally repeat the aforementioned parameter estimation routine five times. We have undertaken this additional step to understand the robustness of the optimization technique used in this work (see Figure 4 and Figure S7 in the Supporting Information). Note that each of these five repetitions will provide an optimal model parameter vector θ*. However, in Table 2 we report only the model parameter vector for the different adsorbents for the repetition that corresponds to the lowest objective function value out of these five repetitions.

## 4 Results

### 4.1 Material Characterization

**Figure 2:**
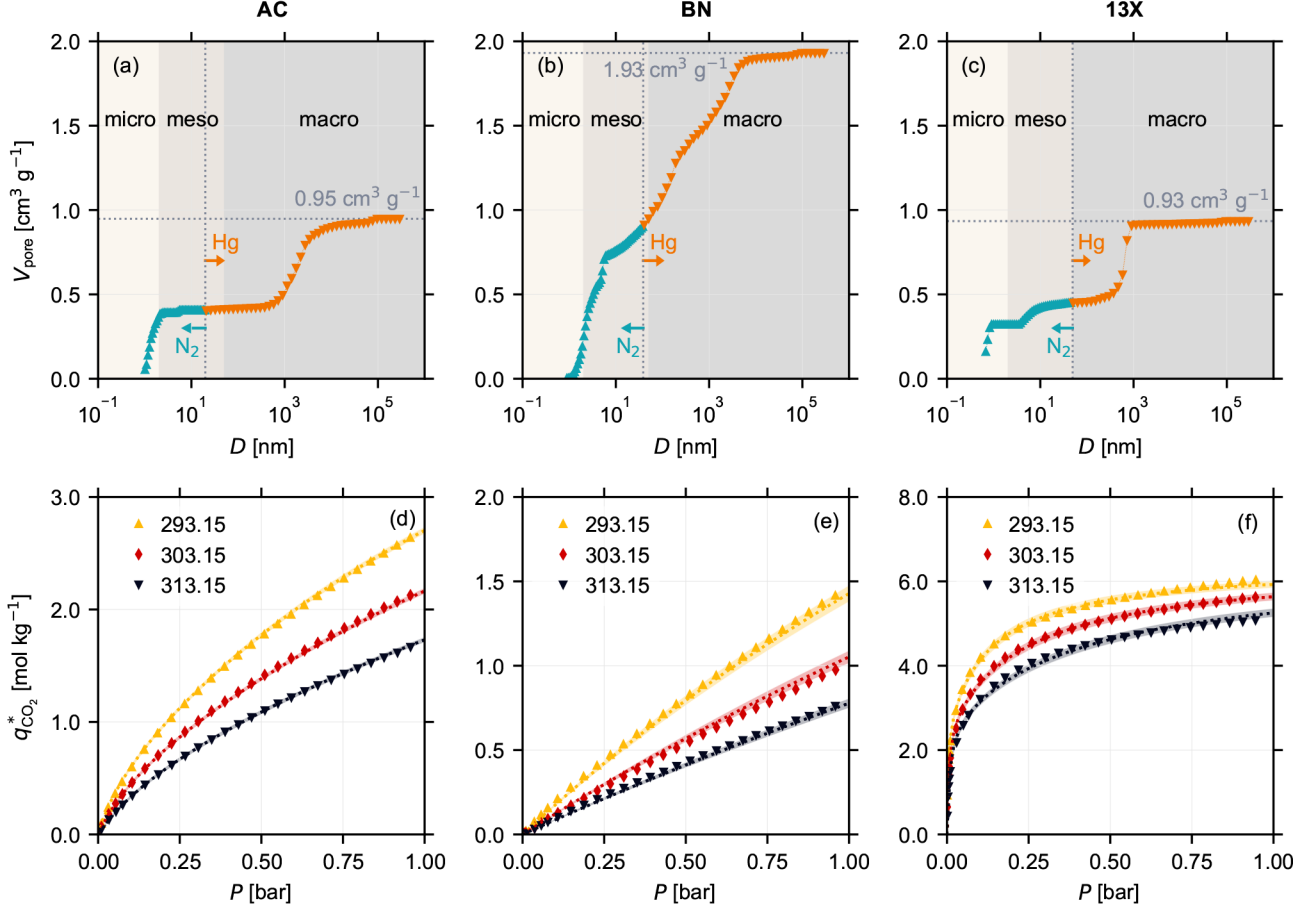
Cumulative pore volume *V*_pore_ as a function of pore diameter *D* for (a) Norit RB3 (AC), (b) boron nitride (BN), and (c) Zeolite 13X (13X) pellets. The cumulative pore volume is obtained using N_2_ adsorption measurements at 77 K (teal) and mercury intrusion porosimetry (orange) on pellets of the three materials. Equilibrium isotherms *q*_CO_2__^*^(*P*, *T*) for CO_2_ estimated at 293.15 K (yellow), 303.15 K (red), and 313.15 K (black), using a commercial volumetric apparatus, for (d) Norit RB3 (AC), (e) boron nitride (BN), and (f) Zeolite 13X (13X) pellets. The curves represent the modeled equilibrium isotherms obtained by fitting the experimental data points to a single-site Langmuir isotherm model for boron nitride and to a dual-site Langmuir isotherm model for Norit RB3 and Zeolite 13X, given by eq 2. The corresponding 95% confidence bounds, obtained using the methodology outlined in Section S4 in the Supporting Information, are highlighted as the shaded region alongside the modeled isotherms. The textural properties and isotherm parameters estimated from these measurements for the three materials are provided in Table 1.

The porosity characterization, given by the cumulative pore volume *V*_pore_ as a function of the pore diameter *D*, for the three materials is shown in Figure 2a–c. The raw N_2_ sorption and MIP data for all three adsorbents is shown in Figure S1 in the Supporting Information. Also, a discussion on the crystallinity of the shaped forms of all the three adsorbents is provided in Section S1 in the Supporting Information. We classified the cumulative pore volume into micro-, meso-, and macropores based on the pore diameter.^18^ We can make three key observations from these results. First, the micropores contribute to the cumulative pore volume for all three adsorbents. Second, the contribution of mesopores to the total pore volume is negligible for AC (panel (a)), indicated by the constant pore volume over the entire range of mesopore diameters, while a greater contribution is seen in 13X (panel (c)) followed by BN (panel (b)). Finally, for all three adsorbents, the macropores contribute the most to the cumulative pore volume. Note that, for materials in their pelletized form, the combination of N_2_ sorption measurements and MIP enables accessing micro-, meso-, and macropores (see vertical dotted lines in panels (a)–(c)). Therefore, characterizing the porosity solely via N_2_ sorption would lead to a significant underestimation of the total pore volume. Additionally, an incomplete pore volume characterization will also lead to an inaccurate estimation of the skeletal density, given by eq 1a. We perform a thorough analysis on the impact of inaccurate porosity characterization on the predicted adsorption behavior, and this analysis is discussed in detail in Section S7.3 in the Supporting Information. The porosities and skeletal densities for the three adsorbents, estimated using eqs 1b and 1a, are provided in Table 1.

**Table 1:** Parameters for the Equilibrium CO_2_ Isotherm, Textural Properties, and Other Variable(s) for Norit RB3 (AC), Boron Nitride (BN), and Zeolite 13X (13X) Used in This Work^a^

		value		
parameter	unit	AC	BN	13X
Isotherm Parameters				
*q*_sb,CO_2__	mol kg^–1^	6.73 ± 0.03	7.01 ± 0.09	2.56 ± 0.03
*b*_0,CO_2__	(×10^–7^) m^3^ mol^–1^	4.13 ± 0.02	2.30 ± 0.03	53.69 ± 5.78
–Δ*U*_b,CO_2__	kJ mol^–1^	25.11 ± 0.01	24.87 ± 0.04	34.94 ± 0.27
*q*_sd,CO_2__	mol kg^–1^	0.48 ± 0.01	-	3.83 ± 0.04
*d*_0,CO_2__	(×10^–7^) m^3^ mol^–1^	13.29 ± 0.79	-	0.13 ± 0.01
–Δ*U*_d,CO_2__	kJ mol^–1^	30.24 ± 0.15	-	40.00 ± 0.09
Textural Properties				
*V*_bulk_	cm^3^ g^–1^	1.54	2.42	1.18
*V*_pore_	cm^3^ g^–1^	0.95	1.93	0.93
ϵ_T_	-	0.61	0.80	0.79
ρ_s_	g cm^–3^	1.68	2.04	4.08
Other Variables				
*m*_ads_	mg	62.5	79.7	59.4

a The equilibrium CO_2_ isotherm parameters have been estimated by fitting experimental data obtained from a commercial volumetric apparatus using the procedure outlined in Section 2.2.2. The corresponding 95% confidence intervals, obtained using the methodology outlined in Section S4 in the Supporting Information, are also reported alongside the estimates.

The volumetric equilibrium CO_2_ isotherms for the three materials at three different temperatures as a function of their partial pressure *q*_CO_2__^*^(*P*, *T*) are shown in Figure 2d–f. We can make two observations. First, the adsorption behaviors in the three materials exhibit contrasting characteristics in terms of the nonlinearity of the isotherm and the total adsorption capacity. On the one end, BN (panel (b)) exhibits a near linear isotherm with the lowest adsorption capacity. On the other end, 13X (panel (c)) exhibits a strongly nonlinear isotherm with the highest adsorption capacity. Finally, AC (panel (a)) exhibits an intermediate behavior in terms of both nonlinearity and adsorption capacity. Second, the isotherm model fits (solid lines) for all the three materials exhibit excellent agreement with the experimental data (markers). Additionally, on the basis of the narrow confidence intervals (relatively small when compared to the estimated parameter values) of the estimated parameters, provided in Table 1, and also visualized by the shaded region alongside the modeled isotherms in panels (d) through (f), we can safely assume that the model parameters for all three adsorbents are well-determined. Note that, for AC and 13X, we used a DSL model, and for BN, we used a SSL model.

We will use the fitted isotherm parameters presented here as references to subsequently validate the outcomes presented in Section 4.2 to simultaneously estimate adsorption equilibria and kinetics using the dynamic gas sorption experiments.

### 4.2 Simultaneous Estimation of Adsorption Equilibrium and Kinetics

**Figure 3:**
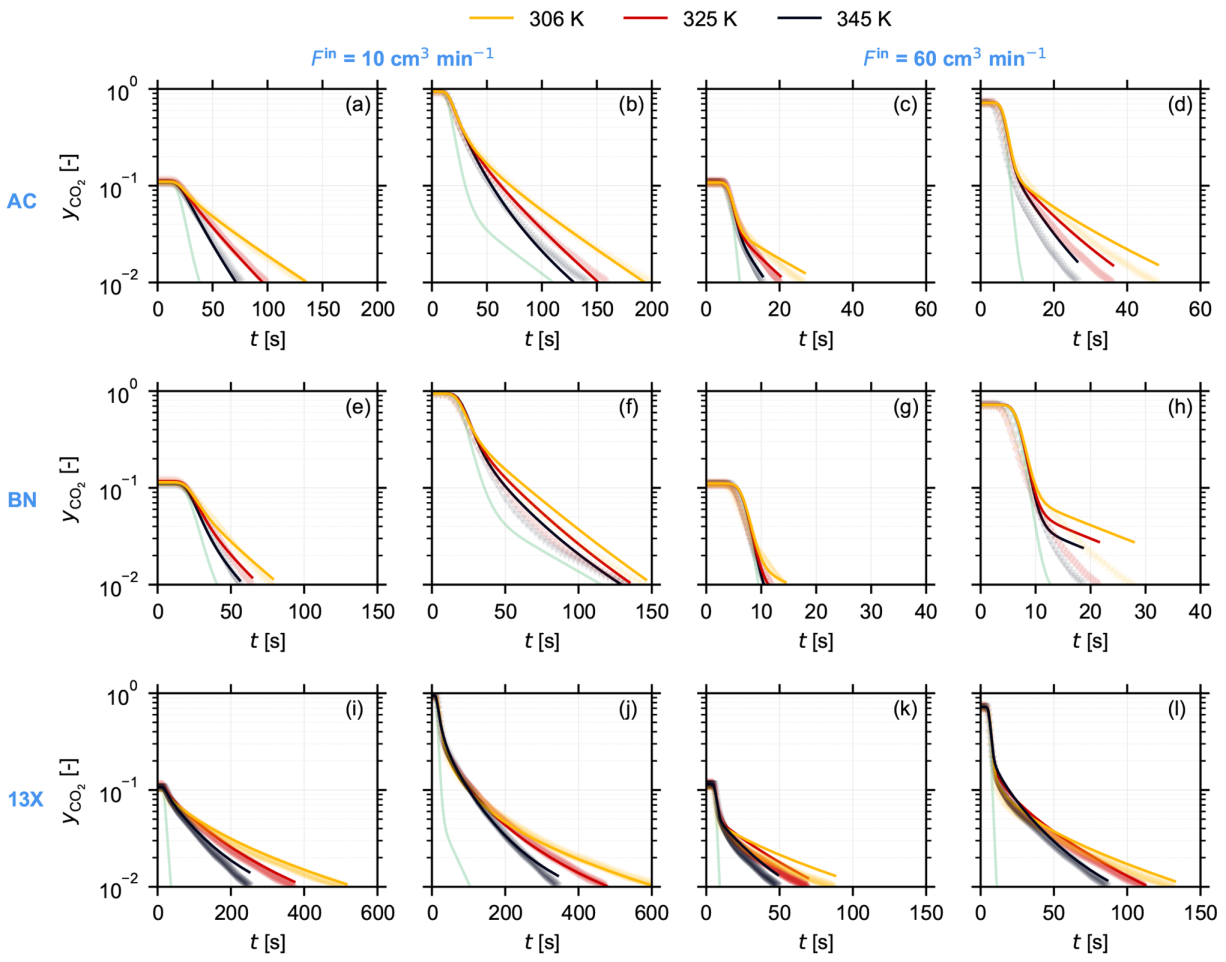
Experimental and simulated desorption responses for Norit RB3 (AC, a–d), boron nitride (BN, e–h), and Zeolite 13X (13X, i–l) pellets at 306 K (yellow), 325 K (red), and 345 K (black): *F*^in^ = 10 cm^3^ min^–1^ and (a, e, i) *y*_0_ = 0.12 and (b, f, j) *y*_0_ = 0.94; *F*^in^ = 60 cm^3^ min^–1^ and (c, g, k) *y*_0_ = 0.11 and (d, h, l) *y*_0_ = 0.73. The markers represent the time evolution of the experimental CO_2_ composition *y*_CO_2__. The solid curves indicate the corresponding simulated response generated using the model described in Section 3.2 with model parameters given in Table 2. The light green curve in each panel represents the blank response of the setup at the given flow rate and initial gas phase composition.

The adsorption isotherm and kinetic parameters along with their confidence intervals, computed at a confidence level η = 0.95, for all three materials, obtained by performing a full curve fit on the experiments using the simulation framework described in Section 3.3, are provided in Table 2. Note that the methodology to characterize the blank volume of the setup, which is a key prerequisite to accurately quantify the adsorption behavior in adsorbents using the proposed methodology, is described in detail in Section S5 in the Supporting Information. The time-resolved experimental CO_2_ gas composition *y*_CO_2__ for the 12 experiments performed at four initial saturation partial pressures, two inert gas flow rates, and three temperatures, along with the corresponding model fit, using the parameters provided in Table 2, for all the materials is provided in Figure 3. We can make several observations based on both the experimental and the simulation outcome that are common for all three materials. First, as expected, the experiment performed at lower temperature takes longer to reach a given gas phase composition at a given flow rate than an experiment performed at higher temperature, indicating a higher adsorption capacity at a lower temperature. Second, we can observe a significant time lag between the blank response (shown in light green) and the experimental response with the adsorbent. Third, even though not explicitly obvious here, by performing experiments at the two chosen flow rates, we have explored both the equilibrium and the kinetic controlled regimes (see Figure S6 in the Supporting Information). When comparing the time-resolved experimental and fitted responses for the three materials, we can make several observations. First, the experiment performed using 13X takes the longest to reach the lowest CO_2_ gas composition, followed by AC and BN. This is in line with the adsorption capacities reported for the three materials in Section 2.2. Second, the overall fit of the simulated response is in good agreement with the experimental responses for AC and BN through the entire composition range. However, for 13X, the agreement is relatively poor (also note the semilog scale for all the plots and the differences in the time scales for each material). We will revisit the cause and implications of this result in the discussion that follows. Additionally, for all the experiments, we have verified their repeatability, and we have provided a discussion in Section S6.1 in the Supporting Information.

**Table 2:** Parameters for the Equilibrium CO_2_ Isotherm and CO_2_ Kinetics on Norit RB3 (AC), Boron Nitride (BN), and Zeolite 13X (13X) from the Dynamic Sorption Experiments, Using the Approach Presented in Section 3^a^

		value		
parameter	unit	AC	BN	13X
Isotherm Parameters				
*q*_sb,CO_2__	mol kg^–1^	8.39 ± 0.39	3.12 ± 0.19	0.81 ± 0.05
*b*_0,CO_2__	(×10^–7^) m^3^ mol^–1^	0.39 ± 0.02	11.62 ± 0.73	91.29 ± 5.70
–Δ*U*_b,CO_2__	kJ mol^–1^	19.88 ± 0.81	22.99 ± 0.82	38.04 ± 2.19
*q*_sd,CO_2__	mol kg^–1^	2.68 ± 0.10	-	4.75 ± 0.25
*d*_0,CO_2__	(×10^–7^) m^3^ mol^–1^	17.36 ± 0.84	-	26.01 ± 1.52
–Δ*U*_d,CO_2__	kJ mol^–1^	25.96 ± 0.27	-	28.83 ± 0.63
Kinetic Parameters				
*k*_1,CO_2__	s^–1^	1.02 ± 0.04	0.07 ± 0.00	779.18 ± 49.36
*k*_2,CO_2__	s^–1^	16.79 ± 0.62	831.77 ± 69.51	75.34 ± 4.22

a The corresponding 95% confidence intervals, obtained using the methodology outlined in Section S4 in the Supporting Information, are also reported alongside the estimates. The parameter values correspond to the repetition with the lowest objective function value obtained using the approach described in Section 3.3.2.

**Figure 4:**
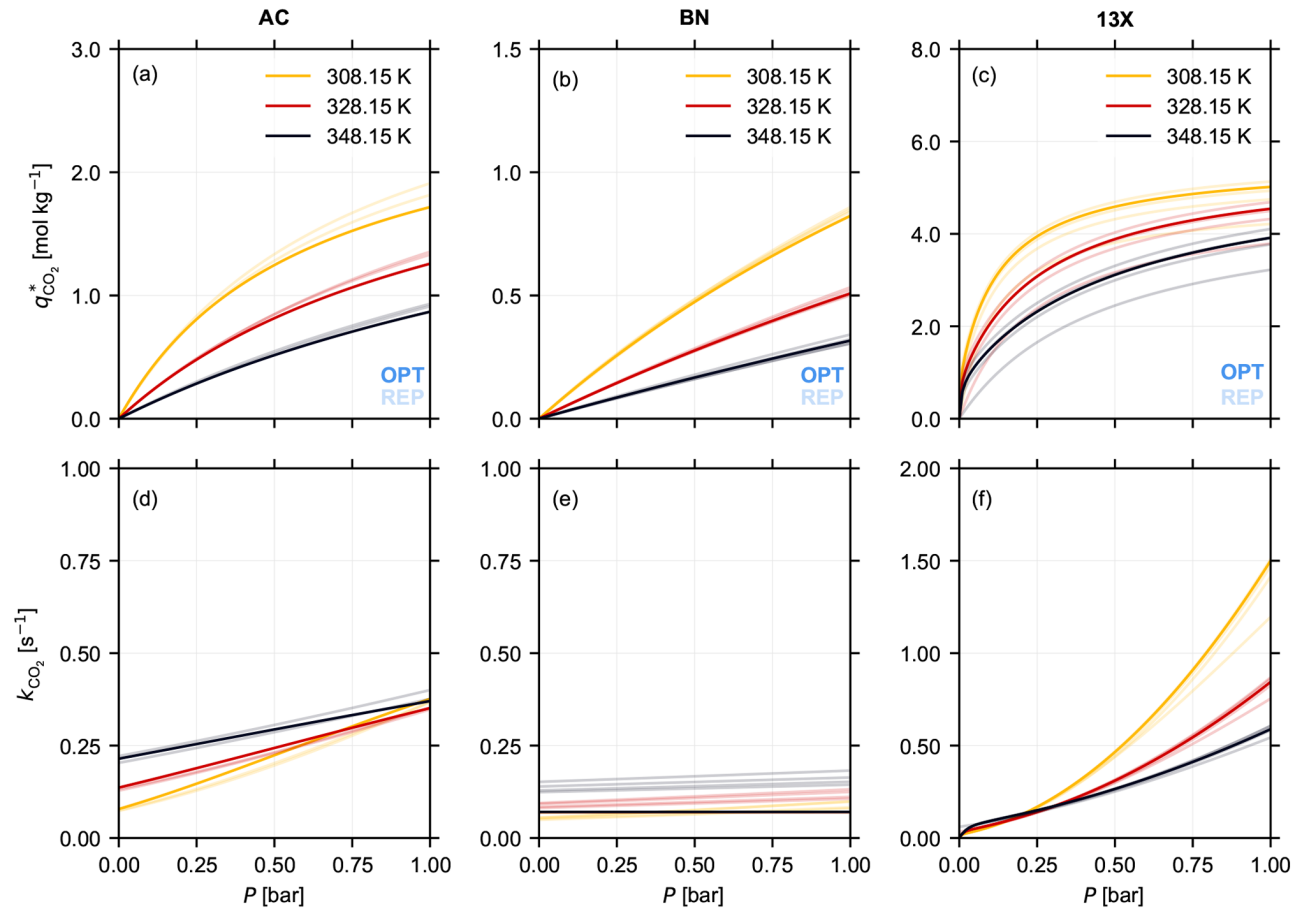
Equilibrium isotherms *q*_CO_2__^*^(*P*,*T*) for CO_2_ estimated at 308.15 K (yellow), 328.15 K (red), and 348.15 K (black), from the dynamic sorption experiments, using the approach presented in Section 3, for (a) Norit RB3 (AC), (b) boron nitride (BN), and (c) Zeolite 13X (13X) pellets. The corresponding lumped kinetic rate constants *k*_CO_2__ for the three materials at the three temperatures are shown in panels (d) through (f). The different curves in the figure represent the isotherms obtained by repeating the parameter estimation procedure, described in Section 3.3.2, five times for each material. The curves with a darker shade (OPT) are obtained using the isotherm parameters given in Table 2, and they correspond to the estimates with the lowest objective function using the approach described in Section 3.3.2. The curves with the lighter shade (REP) correspond to the estimates with a higher objective function.

The CO_2_ isotherms for the three materials at three different temperatures as a function of its partial pressure *q*_CO_2__^*^(*P*, *T*) are shown in Figure 4a–c. The corresponding lumped kinetic rate constants *k*_CO_2__ at the same three temperatures as a function of its partial pressure are shown in Figure 4d–f. Note that the different curves at each temperature indicate the different equilibrium and kinetic estimates obtained by repeating the parameter estimation (REP), as explained in Section 3.3.2. The darker shaded curve corresponds to the estimate with the lowest objective function value obtained from the optimization routine (OPT).

From Figure 4a–c, we can make two key observations on the equilibrium adsorption behavior of CO_2_ on the three materials. First, the equilibrium loading and the shape of the isotherm of the three materials are different. This observation is similar to the isotherms obtained from the volumetric measurement, shown in Figure 2. Second, we can observe differences in the estimated isotherms from the different repeats of the parameter estimation. These differences are smaller for AC (panel (a)) and BN (panel (b)), while for 13X (panel (c)) both the absolute capacity and the shape of the isotherm are vastly different for the different repeats. We attribute this error to the robustness of the optimizer, and we present a thorough analysis of these differences in Section 5.2.

From Figure 4d–f, we can make two key observations on the kinetic behavior of CO_2_ on the three materials. First, similar to the adsorption capacity, the kinetic behaviors, i.e., the temperature and partial pressure dependence of the kinetics, are different for the three temperatures. The optimal solutions (shown in darker shade) for AC (panel (d)) and 13X (panel (f)) exhibit a temperature and partial pressure dependence, while that for BN (panel (e)) does not exhibit either of them. From the kinetic parameter values reported in Table 2 and from eq 6, we can infer that, for AC and 13X, macropore or combined micro/macropore resistance is dominant. Independent measurements reported in the literature have attributed macropore resistance to be dominant in both these adsorbents.^8,34^ However, for BN, we can infer that the micropore resistance is the dominant one. In more detail, for AC and 13X, the contribution from the first term in eq 6 is small or negligible. Therefore, the temperature and partial pressure dependence of the kinetics depends on the second term and, more specifically, on the local slope of the isotherm at those conditions. Second, similar to the adsorption capacity, we can observe differences in the estimated kinetic behavior from the different repeats of the parameter estimation. The absolute kinetic rate constant and the trends for AC and 13X are similar. However, for BN there are repeats of the parameter estimation, with a higher objective function value, that point to a temperature and a small partial pressure dependence of the kinetics. We can attribute these differences to factors arising from computational robustness and not from any underlying physics. This particular case highlights that these measurements can provide an excellent starting point to gauge the kinetics of a system but cannot be used to pinpoint with certainty the controlling mechanism. We can circumvent this issue by repeating the experimental measurements with another inert gas, e.g., argon, as has been proposed elsewhere.^37^ Note that the confidence intervals of the estimated parameters, provided in Table 2, are narrow (relatively small when compared to the estimated parameter values) but are wider than the ones obtained from the volumetric approach presented in Section 4.1.

**Figure 5:**
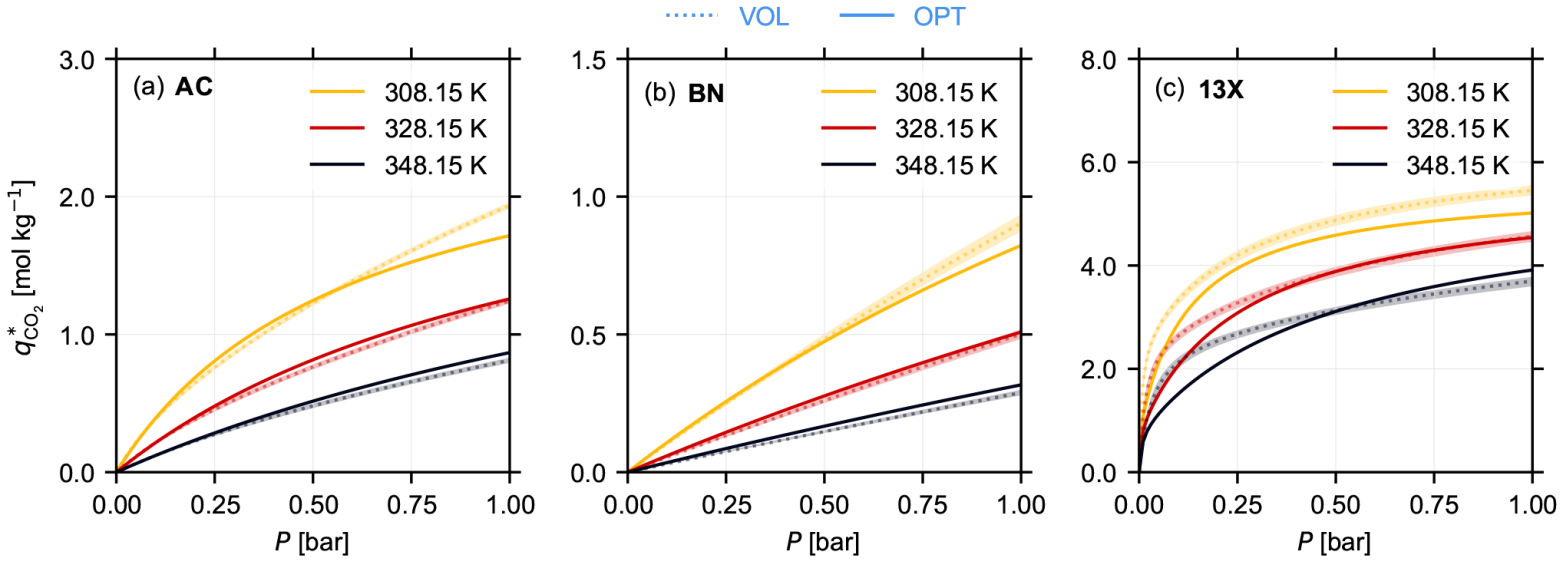
Equilibrium isotherms *q*_CO_2__^*^(*P*, *T*) for CO_2_ estimated at 308.15 K (yellow), 328.15 K (red), and 348.15 K (black) for (a) Norit RB3 (AC), (b) boron nitride (BN), and (c) Zeolite 13X (13X) pellets. The curves with the dotted lines (VOL) correspond to the estimates obtained by fitting isotherm models to volumetric measurements discussed in Section 4.1 and Figure 2. The corresponding 95% confidence bounds, obtained using the methodology outlined in Section S4 in the Supporting Information, are highlighted as the shaded region alongside the isotherms from the volumetric measurements. The curves with the solid lines (OPT) are obtained using the isotherm parameters given in Table 2, and they correspond to the estimates with the lowest objective function from the dynamic sorption experiments, using the approach described in Section 3.

The preceding discussion does not provide a quantitative validation of the obtained isotherm estimates. Therefore, we compared the aforementioned equilibrium isotherms with the ones obtained from the volumetric measurements, presented in Section 4.1. The CO_2_ isotherms for the three materials at three different temperatures as a function of their partial pressure *q*_CO_2__^*^(*P*, *T*) obtained from the dynamic sorption experiments (OPT) and the volumetric method (VOL) are shown in Figure 5. We can make two key observations. First, there is an excellent agreement between the two techniques over a wide range of temperatures and partial pressures for AC (panel (a)) and BN (panel (b)). This is highlighted by the predicted isotherms falling within the confidence regions obtained from the volumetric measurements for most partial pressures and temperatures for these two materials. At high partial pressures and low temperatures, there is a small error in the estimated adsorption capacity from the dynamic sorption experiments. We attribute this error to the robustness of the optimizer, and we present a thorough analysis on these differences in Section 5.2. Second, for 13X, even though the shape of the isotherm is captured to a reasonable accuracy, there is a significant discrepancy between the two techniques at lower partial pressures. We attribute this error to two factors, namely, to the robustness of the optimizer and to the gas composition estimation from the MS. For 13X, unlike AC and BN, the slope of the isotherm at very low partial pressures (<0.05 bar) is very steep. Therefore, to capture the shape of the isotherm and thereby the adsorption capacity accurately, at these low partial pressures we should have a good gas phase composition resolution. However, the lowest CO_2_ partial pressure we can reliably measure with our gas detector was 0.01 bar (see discussion presented in Section S2.4 in the Supporting Information). Therefore, we can expect to observe a discrepancy between the two techniques. We can circumvent this issue either by employing a more sensitive gas detector or by performing a simple mass balance, forgoing the full curve fit, to obtain the adsorption capacity at high partial pressures.^35^ Finally, note that we cannot quantitatively validate the estimated kinetic rate constants due to the unavailability of an independent commercial measurement technique.

## 5 Discussion

### 5.1 Contextualization and Contributions

Studies looking into techniques for rapid experimental characterization of adsorption behavior have gained momentum over the past few years. Several techniques that use commercial, modified commercial, or purpose-built adsorption equilibrium and kinetics characterization devices using small amounts of adsorbent (on the order of few 10s or 100s of milligrams) have been reported. Some of these techniques also enable studying multicomponent systems.^27,29,35,47,48^ Driven by this momentum, we have developed the material characterization pipeline presented in this work to use less than 100 mg of a given adsorbent to obtain both the textural and the adsorption properties.

The approach used to develop the technique presented in this work was inspired by dynamic gas sorption techniques, namely, ZLC and DCB. The ZLC method is designed to use small amounts of adsorbent (milligram scale) to study diffusion behavior in porous materials, often at low partial pressures.^36^ It is also designed to have negligible extra column volumes from fittings, gas lines, and sensors. This is necessary because, when using small amounts of adsorbent, one cannot neglect the extra column volumes as this will be comparable to the volume of gas adsorbed, thereby making it a necessity to explicitly account for these using a model. The DCB method is designed to use large amounts of adsorbent (gram to kilogram scale) to study dynamics of gas sorption in a packed bed column. Unlike the ZLC, in most cases, it is difficult to completely eliminate extra column volumes in DCB setups. Therefore, it is explicitly modeled, and the output responses are corrected to obtain the *true* column responses.^33^

Our approach exploits the best of both the aforementioned methods. We use small amounts of adsorbent, like the ZLC method, thereby leading to a comparable mathematical framework to describe gas sorption. Yet, we have also modeled the extra column volumes, like the DCB method, thereby forgoing the need to completely eliminate it or minimize it, as in the case of the ZLC method. The aforementioned standard methods and our method share much of the design philosophy, both experimentally and computationally. Despite the number of similarities with the ZLC and DCB setups, we have made a number of new contributions on both the experimental and the computational aspects. The approach presented here will make estimating adsorption equilibria and kinetics more accessible to other material chemists and process engineers. Some of these key contributions are

**Experimental Contributions**
- Providing an integrated material characterization framework that aims to characterize the textural properties, e.g., skeletal density and porosity, which is then subsequently used to characterize adsorption equilibrium and kinetics (see Sections 2.2 and 3.1)
- Designing and building a dynamic gas sorption experimental setup, that uses small amounts of adsorbent (<100 mg), without the need to invest significant effort to minimize blank volumes (see Section 3.1)
- Performing experiments at a broad range of partial pressures, i.e., not limited to dilute conditions (see Section 3.1.2)
- Elucidating the need to perform a thorough calibration of the gas composition detector to obtain accurate estimates of equilibrium and kinetics, especially when using small amounts of adsorbent (see Section S2.4 in the Supporting Information).

**Computational Contributions**
- Defining the rate of gas sorption in the adsorbent using a linear driving force model to enable easy integration of kinetics into detailed process models (see *Gas Adsorption in the Adsorbent*, Section 3.2.1)
- Providing a generic modeling framework to accurately characterize blank volumes present in a dynamic gas sorption experimental setup (see *Blank Volume*, Section 3.2.2)
- Providing a robust parameter estimation methodology, using statistically sound techniques, to obtain adsorption equilibrium and kinetic parameter estimates and their corresponding confidence bounds by performing full desorption curve fits (see Section 3.3.2)
- Developing a digital twin of the experimental setup to study the impact of model inputs (e.g., blank volume model, porosity), parameter estimation algorithms, and operating conditions and to highlight the robustness of the entire material characterization pipeline (see Section 5)
- Providing the computational tools developed in this work through a dedicated open-source platform for future practitioners (see https://github.com/ImperialCollegeLondon/ERASE)

### 5.2 Evaluation of Accuracy Using a Digital Twin

To gain better understanding of the methodology proposed in this work, we have developed a digital twin of our experimental setup. This digital twin coupled with the parameter estimator, described in Section 3.3.2, forms the core of the computational test bench used in this section to perform several analyses.

In this work, the digital twin is described by the mathematical models to represent adsorption behavior and experimental blank volume, presented in Section 3.2, but with known model parameters. The key inputs to the digital twin are the operating conditions (i.e., partial pressure, temperature, and flow rate of the gas), the textural characteristics, and the adsorption and the blank volume model parameters. Using the values provided in Table S3 in the Supporting Information and following the steps provided in Section 3.3.1 to simulate a real experiment, we computationally generate time-resolved responses, similar to the experimental response shown in Figure 3. The 12 computationally generated responses for each material at four initial saturation partial pressures, two inert gas flow rates, and three temperatures, from the digital twin are visualized in Figure S7 in the Supporting Information. We assume this computationally generated response to serve as a proxy for the experimental responses. This assumption has one big advantage for the subsequent discussion, i.e., there are no errors arising from the components of the experimental setup. Similar to the methodology discussed in Section 4.2 with the experimental responses, we feed the computationally generated responses to the parameter estimator, described in Section 3.3.2, to obtain the adsorption equilibria and kinetic parameters.

The digital twin provides us with an idealized *in silico* environment to probe the choices we have made in this work. To this aim, we perform an in-depth analysis on the robustness of the simulation framework, specifically the parameter estimator and on the sensitivity of the model inputs, e.g., porosity and blank volume model, to accurately estimate the properties of interest.

#### 5.2.1 Robustness of the Simulation Framework

In this first study, we want to understand the robustness of the simulation framework. To this aim, we perform a full curve fit of the computationally generated responses, using the simulation framework described in Section 3.3, to obtain the isotherm and kinetic parameters. Note that, for the full curve fit, we use the same textural characteristics and blank volume model parameters as the one used to generate the computational response. Therefore, ideally, if the optimizer is robust, the estimated equilibrium and kinetic parameters should be equal to the corresponding parameters used to generate the computational responses, given in Table S3 in the Supporting Information.

**Figure 6:**
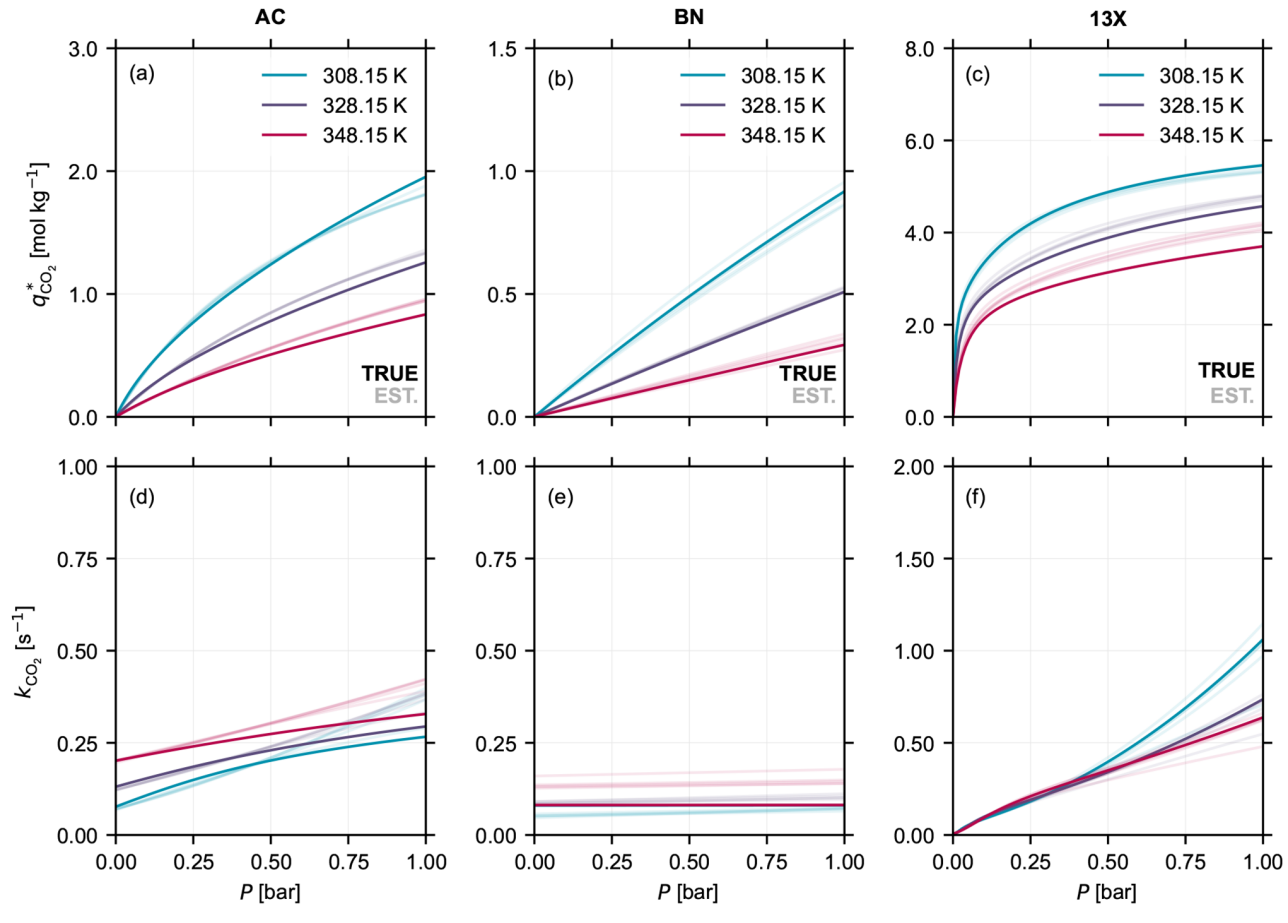
Equilibrium isotherms q*(*P*, *T*) for CO_2_ estimated at 308.15 K (teal), 328.15 K (blue), and 348.15 K (rose), using the approach presented in Section 5.2, for (a) Norit RB3 (AC), (b) boron nitride (BN), and (c) Zeolite 13X (13X) pellets. The corresponding lumped kinetic rate constants *k*_CO_2__ for the three materials at the three temperatures are shown in panels (d) through (f). The curves with a darker shade (TRUE) correspond to the curves obtained using the equilibrium and kinetic parameters of the digital twin, given in Table S3 in the Supporting Information. The curves with the lighter shade (EST.) correspond to estimates obtained by performing parameter estimation on the computationally generated responses. The different curves with a lighter shade are obtained by repeating the parameter estimation procedure, described in Section 3.3.2, five times for each material.

The CO_2_ isotherms for the three materials at three different temperatures as a function of their partial pressures *q*_CO_2__^*^(*P*, *T*), obtained from the full curve fit are shown in Figure 6a–c. The corresponding lumped kinetic rate constants *k*_CO_2__ at the same three temperatures as a function of their partial pressures are shown in Figure 6d–f. Note that the different light shaded curves at each temperature indicate the different equilibrium and kinetic estimates obtained by repeating the parameter estimation (EST.), as explained in Section 3.3.2. The darker shade curve corresponds to the estimate obtained by using the parameter values used to generate the computational response (TRUE). From Figure 6, we can make three key observations. First, there are differences in the estimated isotherms (panels (a)–(c)) from the different repeats of the parameter estimation for the three materials, and they do not overlap with the TRUE isotherm. The former point is in line with the observations made in Section 4.2. Second, for Zeolite 13X, unlike the experimental estimation shown in Figure 5c, the shape of the isotherm and the absolute equilibrium capacity over a broad range of partial pressures and temperatures is closer to the TRUE isotherm. Therefore, the deviation in the experimental isotherm can only arise from a systematic error in the experimentally obtained response. In more detail, we cannot rule out errors in the composition determined from the MS at long times (see discussion presented in Section S2.4 in the Supporting Information), and for 13X this effect is amplified by the longer tail at lower compositions. This is visualized for the different responses observed in the experimental (see panels (e)–(h) of Figure 3) and the computational case (see panels (e)–(h) of Figure S8 in the Supporting Information). Third, similar to the isotherms, we can observe differences in the estimated kinetic behavior, i.e., the temperature and partial pressure dependence of kinetics (panels (d)–(f)). The case of BN is of particular interest. The TRUE kinetic behavior corresponds to a scenario analogous to micropore resistance, i.e., constant kinetic rate constant at all temperatures and partial pressures. However, for a few repeats, the estimated kinetics exhibit temperature dependence and small partial pressure dependence. This is similar to the observations from the experimental estimates for BN.

We further analyzed the objective function values *J*(θ), given by eq S2 in the Supporting Information, obtained at the local minimizer θ* for the five repeats of the parameter estimation for all the three materials. This is visualized in Figure S7 in the Supporting Information. We compare these values with the *true* objective function *J*_true_ value obtained by evaluating it with adsorption equilibrium and kinetic parameters used to generate the computational responses. On the basis of the comparison, we can conclude that the optimizer in the idealized setting described in this section does not manage to reach the optimal parameter values. This is reflected by the higher values of objective function when compared to the *true* objective function *J*_true_. Therefore, the deviations that we observe here between the EST. and the TRUE isotherms or kinetic rate constants are inevitable. This issue can be circumvented by tuning the settings of the optimization algorithm, i.e., genetic algorithm, or changing the optimization algorithm altogether. However, within the scope of this work, we have not invested further effort to resolve this. Note that the absolute equilibrium capacity and the kinetic rate constant from experiments and the digital twin are similar, if not the same. Therefore, the analysis performed here can be used to explain the observations from the experimental runs presented in Section 4.2.

To summarize, we can conclude on the basis of the analysis presented here that the parameter estimation framework used in this work is robust enough to provide a good estimate of the adsorption equilibria and kinetic parameters for all three materials. However, if one obtains poor quality experimental data in regions that have a significant impact on parameter estimation, like low compositions for 13X, a deterioration in the robustness of the parameter estimator is unavoidable.

#### 5.2.2 Sensitivity to Model Inputs

In addition to understanding the robustness of the simulation framework, we wanted to study the sensitivity of the model inputs, e.g., porosity and blank volume model, to accurately estimate the adsorption equilibria and kinetics of the materials. To this aim, we carried out further analysis using the digital twin. The methodology and the obtained results are presented in Section S7.3 in the Supporting Information.

### 5.3 Key Limitations

We acknowledge that there are a few limitations of the work presented here, and these limitations will be addressed in our future work. First, we have performed all the studies in this work using the pelletized form of the adsorbent. To show generality of the complete framework, one should use the characterization pipeline for powders and monoliths. Second, we do not observe a high degree of agreement in the adsorption equilibrium between the dynamic gas sorption and the volumetric measurements for highly nonlinear materials (e.g., 13X), due to the low accuracy of the gas detector at lower compositions. To tackle this, one can use either a specialized detector for a given gas or a combination of detectors sensitive at low and high compositions to ensure high accuracy in the entire range of compositions of interest. Third, we have shown the framework to be robust for a unary system at subatmospheric and atmospheric partial pressures, but systems at high pressures and multicomponent systems have not been studied yet. Fourth, even though we have estimated a lumped kinetic rate constant, using the experimental methodology presented here, we can only infer the controlling mass transfer resistance. To pinpoint the controlling resistance with certainty, one must alter the experimental methodology to include additional experiments with another carrier gas. Finally, and most importantly, the computational time required to perform the parameter estimation is comparable to the time taken to undertake all the experiments for a given adsorbent. This could be easily circumvented by exploiting advances in machine learning to speed up the parameter estimation by replacing the detailed model with a surrogate model.

## 6 Concluding Remarks

The work presented here provides a unified characterization pipeline to describe the textural and adsorption properties of individual shaped adsorbents. To this aim, for the former, we have used a combination of two commercially available techiniques, i.e., N_2_ sorption at 77 K and mercury intrusion porosimetry. For the latter, we have developed a dynamic sorption experimental setup and complemented it with detailed mathematical models. These models serve two purposes: first, to simultaneously estimate the adsorption equilibrium and kinetic parameters by wrapping a parameter estimator around the models, and second, to analyze the robustness of the simulation framework and the impact of various input variables to the model on the prediction accuracy of the adsorption behavior by using the modeling framework as a digital twin. The key outcomes from this work can be summarized as follows:

- One can quantify the unary adsorption equilibria and kinetics across a wide range of temperatures and partial pressures using a small quantity of material (<100 mg) in a matter of few hours, making it ideally suited for newly synthesized porous materials.
- One should take utmost care in characterizing the textural characteristics and the blank volume in dynamic sorption experimental setups when using a small quantity of material to accurately describe the adsorption behavior.
- One should carefully select the gas composition detector in the experimental setup and the optimizer in the parameter estimator to eliminate systematic errors in the estimated parameters to quantitatively describe adsorption behavior.

## Supporting Information Available

Additional information on the textural characterization of the materials; data preprocessing; maximum likelihood estimation and uncertainty calculation; experimental framework; characterization of the blank volume; and experimental and computational studies for the estimation of equilibrium and kinetics (PDF)

## Notes

All the raw data, processed data, and simulation results generated for the work presented in this article are available through 10.48420/19781164. The MATLAB and Python scripts for the analysis of the experiments and for the mathematical models in the computational framework are available through https://github.com/ImperialCollegeLondon/ERASE.
