# Supplementary material for: Simultaneous Estimation of Gas Adsorption Equilibria and Kinetics of Individual Shaped Adsorbents
Source: Chem Mater. 2022 Jul 27;34(15):6671–86. doi: 10.1021/acs.chemmater.2c01567 (PMC9367012; doi:10.1021/acs.chemmater.2c01567)
Supplement: Supplementary file 1 — cm2c01567_si_001.pdf [file cm2c01567_si_001.pdf]

Supporting Information (SI) for "Simultaneous Estimation of Gas Adsorption Equilibria and Kinetics of Individual Shaped Adsorbents"

Hassan Azzan,[†,§] Ashwin Kumar Rajagopalan,[*,†,‡,§] Anouk L'Hermitte,[†,¶] Ronny Pini,[†] Camille Petit[†]

Contents

S1 Textural Characterization of the Materials **2**

S2 Experimental Framework **3**

S2.1 Experimental Setup 3

S2.2 Degassing Protocol 3

S2.3 Design of Drying Columns 4

S2.4 Calibration of Mass Spectrometer 4

S2.5 Calibration of Mass Flow Meter 5

S3 Data Preprocessing **6**

S3.1 Volumetric Adsorption Equilibrium 6

S3.2 Simultaneous Estimation of Adsorption Equilibrium and Kinetics 6

S4 Maximum Likelihood Estimation and Uncertainty Calculation **7**

S5 Characterization of Blank Volume **7**

S5.1 Blank Volume Experiments 7

S5.2 Blank Volume Model 8

S5.3 Estimating Blank Volume Model Parameters 9

S5.4 Characterization of Blank Volume 10

S6 Estimation of Equilibrium and Kinetics: Experimental **11**

S6.1 Repeatability of Experiments 11

S6.2 Flow Dependency on Adsorption Behavior 12

S6.3 Objective Function 13

S6.4 Model Parameter Values and Bounds 15

S7 Estimation of Equilibrium and Kinetics: Computational **17**

S7.1 Model Parameter Values 17

S7.2 Computationally Generated and Fitted Desorption Responses 17

S7.3 Sensitivity to Model Inputs 19

S7.4 Impact of Blank Volume Model 21

[*] Email: ashwinkumar.rajagopalan@manchester.ac.uk

[†] Department of Chemical Engineering, Imperial College London, London, SW7 2AZ, United Kingdom

[‡] Current Address: Department of Chemical Engineering, The University of Manchester, Manchester, M13 9PL, United Kingdom

[¶] Department of Materials, Imperial College London, London, SW7 2AZ, United Kingdom

[§] These authors contributed equally to this work.

S1 Textural Characterization of the Materials

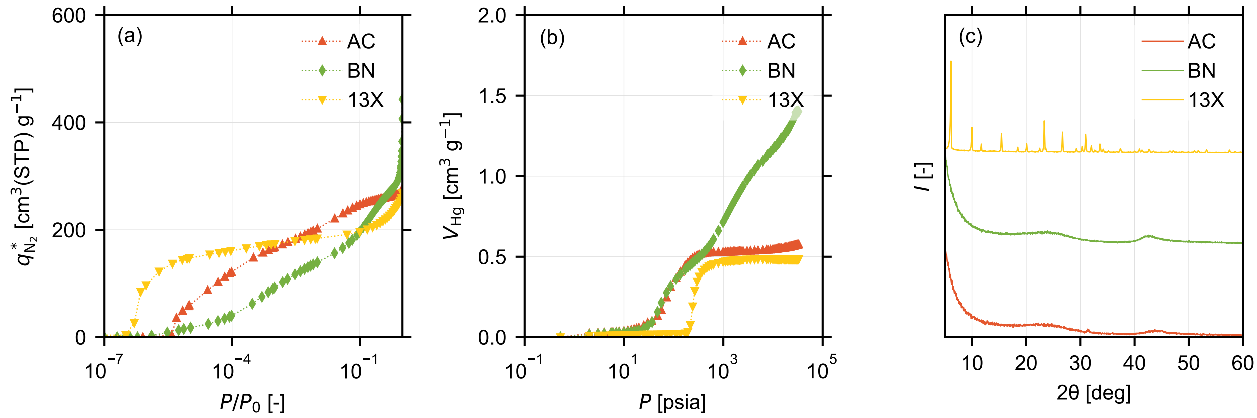

Figure S1: **(a)** N_2 adsorption isotherms measured at 77 K using a Quantachrome Autosorb iQ to characterize the pore size distribution in the micropore region for AC (red), BN (green), and 13X (yellow). **(b)** The corresponding cumulative mercury intrusion against pressure measured using a Micromeritics AutoPore IV Series Mercury Porosimeter to characterize the pore size distribution in the macropore region. The data from panels (a) and (b) is analyzed to obtain the textural characteristics, given in Table 1 of the main text, of the three materials. **(c)** X-ray diffraction patterns obtained using a Malvern Panalytical XRD X'pert PRO to characterize the crystal structure of the adsorbent materials.

In Figure S1a, we illustrate the raw data obtained from the N_2 sorption isotherms at 77 K for all the three materials. We can make several observations and gain insights into the nature of the pore structure within the three materials studied in this work. First, AC and 13X conform to the Type I isotherm classification. For these materials, we can observe a steep uptake of N_2 at low partial pressures followed by a plateau, characteristic of microporous materials.[1] Second, we can observe that the micropore filling occurs at a lower relative pressure (P/P_0) in 13X when compared to AC which corresponds to a decrease in micropore width and an increase in adsorption energy. Additionally, the range of relative pressure required to reach a plateau is smaller in 13X when compared to AC, indicating a limited range of micropore sizes as seen in Figure 2 of the main text. Finally, BN conforms to a Type IV isotherm, as observed elsewhere,[2] characteristic of a mesoporous material. This can also be observed in Figure 2 of the main text.

In Figure S1b, we illustrate the raw data obtained from Mercury Intrusion Porosimetry (MIP) for all the three materials. We can make two observations. First, for AC and BN, the pressure at which intraparticle filling is observed is the lowest among the three materials. This is indicative of a large range of macropore sizes. Second, 13X shows a sharp uptake at a higher pressure when compared to AC, indicating a smaller range of macropore sizes. These observations are also visualized in Figure 2 of the main text.

In Figure S1c, we illustrate the X-Ray diffraction (XRD) patterns for all the three materials. We can make two observations. First, both AC and BN show broad peaks in the ranges 20° to 30° and 40° to 45°, indicative of an amorphous structure. For BN these peaks are characteristic of turbostratic/amorphous BN,[3] which is the form expected to be obtained using the synthesis method we employed in the work.[4] Second, 13X exhibits sharp peaks, indicative of its crystalline structure.

S2 Experimental Framework

S2.1 Experimental Setup

Photographs of the setup are shown in Figure S2. The details of the corresponding components used to assemble the experimental setup is described in Section 3.1 of the main text.

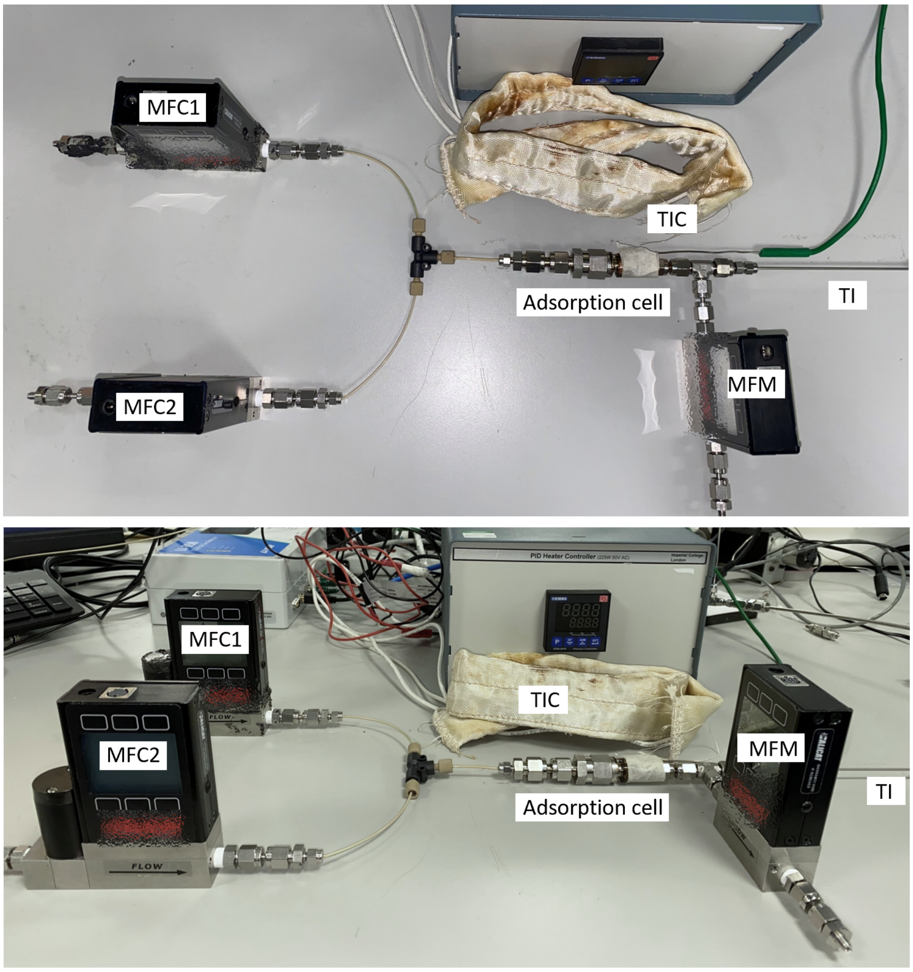

Figure S2: Top and side views of the experimental setup, described in Section 3.1 of the main text and Figure 1 of the main text, excluding the gas cylinders and drying columns (DC) of the gas supply system and the mass spectrometer (MS).

S2.2 Degassing Protocol

For the volumetric and MIP experiments (Section 2.2 of the main text), we degassed the AC and BN *ex situ* in the degassing stations of an Autosorb iQ (Quantachrome Instruments, United States) using the following protocol: (1) 1 h at 323 K; (2) 1 h at 373 K; and (3) 16 h at 393 K. Between the three steps, we employed a temperature ramp at a constant rate of $1\,\mathrm{K\,min^{-1}}$. For the dynamic sorption experiments, we performed a two stage degassing. First, we employed the same degassing protocol as the volumetric experiments. Second, we performed an *in situ* degassing of the adsorbent in the adsorption cell. We kept the adsorbent at 393 K, under constant helium flow at $10\,\mathrm{cm^3\,min^{-1}}$ for at least two hours. This was performed to remove any residual moisture in the rods that may have been adsorbed when transferring it to the adsorption cell. The *in situ* degassing was only performed immediately after the rods were placed in the adsorption cell. For the volumetric and MIP experiments, we degassed 13X using the

following protocol: (1) 1 h at 323 K; (2) 2 h at 373 K; and (3) 16 h at 623 K. Between the three steps, we employed a temperature ramp at a constant rate of $1\,K\,min^{-1}$. For dynamic sorption experiments, we performed a two stage degassing. First, we employed the same degassing protocol as the volumetric experiments. Second, we performed an *in situ* degassing of the adsorbent at 473 K over night before experiments conducted at each of the three temperature studied in this work.

S2.3 Design of Drying Columns

Stainless steel drying columns (DC) (Section 3.1 of the main text) were built in-house and were packed with approximately 5 g of Molecular Sieve, Type 4A beads (Sigma Aldrich, United Kingdom). The DCs were built using 1/2" stainless steel tubing and fittings to ensure good thermal conductivity. We placed the DCs inside a Fisherbrand Isotemp R20 Refrigerated and Heated Bath Circulator (Fisher Scientific, United Kingdom). During dynamic gas sorption experiments, to enhance adsorption of moisture present in the gas from the cylinders, we set the temperature of the bath to 273.15 K. During the *in situ* regeneration of the dessicant, we set the temperature of the bath to 373.15 K at a constant inert gas flow at $10\,cm^3\,s^{-1}$. At regular intervals, we carried out an *ex-situ* degassing of the desiccant by placing the columns in an oven at 473.15 K under constant helium flow. We used these drying columns to avoid obtaining inaccurate experimental responses. This was observed, particularly in strongly hygroscopic materials such as Zeolite 13X, in the presence of moisture in the feed gas even at low compositions (5 ppm), as seen in Figure S5.

S2.4 Calibration of Mass Spectrometer

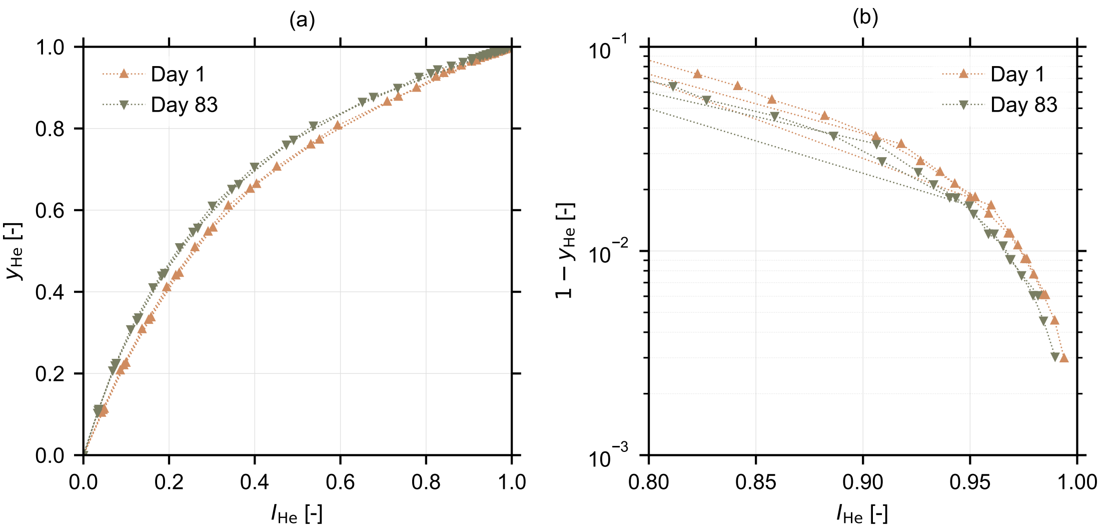

Figure S3: Comparison of calibration plots for binary mixtures of CO_2 and He, carried out at an interval of 83 days. The calibration curve is obtained using the ratio of the MS signal for He to the sum of the MS signal for CO_2 and He (I_{He}). **(a)** Full calibration curve at compositions ranging from pure CO_2 to pure He. **(b)** Calibration curve plotted with CO_2 mole fraction $(1 - y_{He})$ against I_{He}. Note that panel (b) is a semilog plot and the different curves of a given color correspond to the calibration curves obtained at different flow rates.

We calibrated the mass spectrometer (MS) to obtain the gas phase composition of the binary mixture. We performed the calibration by introducing mixtures of different compositions at a constant

total flow rate with an empty adsorption cell. We tracked the He and CO_2 MS signal. To generate mixtures of different compositions, the two gases at different flow rates, determined by the set points of the MFC, were mixed. Finally, to obtain a calibration curve, we fit the known compositions of the gas mixtures y_{He} to the MS signal ratio (I_{He}) of He signal to the sum of He and CO_2 signals. We repeated this calibration at three different total flow rates ($F^{\mathrm{in}} = [10\ 30\ 60]\mathrm{cm^3\,min^{-1}}$) to ensure the variation, if any, in the obtained MS signal due to changes to the total flow rate of the gas mixture is captured. We also performed a repeat of the MS calibration after 83 days of the first calibration. We used an in-built function `fit` with linear interpolation in MATLAB R2020a (The Mathworks Inc., United States) to obtain the calibration curve for the two repeats performed on two different days at different flow rates.

The calibration curve obtained from the two repeats is visualized in Figure S3. Two key observations can be made. First, the calibration curve for both the repeats are nonlinear. Second, there is a significant drift in the calibration between the two repeats. Such a drift in the calibration will have implications on the actual gas phase composition and the shape of the desorption response obtained during a dynamic sorption experiment. For instance, due to the drift, at a signal intensity ratio $I_{\mathrm{He}} = 0.50$, the differences in the esimated gas composition can be as high as $5\,\%$. The implication of this behavior is further stark when observing low CO_2 compositions ($y_{\mathrm{CO_2}} < 0.10$) visualized in Figure S3b. At $I_{\mathrm{He}} = 0.90$, the obtained CO_2 compositions $y_{\mathrm{CO_2}}$ can vary between 0.02 to 0.04. For materials that exhibit a linear or moderatly nonlinear isotherm, such a difference will not have a huge impact on the estimated adsorption properties. However, for materials that exhibit a nonlinear isotherm (like 13X), it is common to observe a long tail at low gas compositions due to the shape of the isotherm (see Figure 3 of the main text and Figure S8). Therefore, an inaccurate estimation of the gas composition would lead to an inaccurate estimation of both adsorption equilibria and kinetics. As stated in the main text, this is one of the possible reasons for the disagreement observed between the equilibrium behaviour predicted for 13X by the approach presented in this work and the volumetric approach. Therefore, it is necessary to repeat MS calibrations at frequent intervals. Additionally, when exploring such broad ranges of compositions, it might be beneficial to use a more sensitive sensor at low gas phase compositions in tandem with a MS.

S2.5 Calibration of Mass Flow Meter

The mass flow meter (MFM) is originally calibrated to a pure gas (in this case He). In order to obtain the mass flow rates of mixtures used in the experiments, we calibrated the MFM to different compositions of He and CO_2 mixtures. To obtain the true mass flow rate of the gas mixtures, we used a universal mass flow meter (UMFM, Agilent ADM flow meter, Model G6691A, Agilent Technologies, United States). To obtain different mixture composition for calibrating the MFM, we varied the flows of the two mass flow controllers (MFC1 for He and MFC2 and CO_2). Subsequently, we mixed the streams and we measured the flow rate using the MFM. The outlet from the MFM was connected to the UMFM to measure the true mass flow rate. We carried out calibration experiments at different total flow rates of the feed mixture, $F^{\mathrm{in}} = [2.0\ 5.0\ 20.0\ 35.0\ 50.0\ 70.0\ 90.0\ 110.0]^{\mathrm{T}}\ \mathrm{cm^3\,min^{-1}}$, where F^{in} is the sum of the flow rate obtained from the two MFCs. For each flow rate, we obtained gas mixtures of varying CO_2 composition y_{in}, ranging from pure He to pure CO_2. Finally, to obtain the actual mass flow rate F_{act}, we fit the resulting flow rate measured by the UMFM F_{true} to the flow rate measured by the MFM F_{MFM}, using a 3rd order polynomial, and CO_2 gas composition y_{in}, using a 2nd order polynomial. We used an in-built function `fit` in MATLAB R2020a (The Mathworks Inc., United

States) to obtain the following polynomial expression that provides the actual mass flow rate F_{act} of the mixture

$$\begin{aligned}F_{\mathrm{true}} =& -0.123 - 0.395 y_{\mathrm{in}} + 1.048 F_{\mathrm{MFM}} - 0.824 y_{\mathrm{in}}^2 + 0.085 y_{\mathrm{in}} F_{\mathrm{MFM}} + \\ & 1.104 \times 10^{-4} F_{\mathrm{MFM}}^2 + 0.240 y_{\mathrm{in}}^2 F_{\mathrm{MFM}} + 5.513 \times 10^{-4} y_{\mathrm{in}} F_{\mathrm{MFM}}^2 - 1.674 \times 10^{-6} F_{\mathrm{MFM}}^3\end{aligned} \tag{S1}$$

S3 Data Preprocessing

S3.1 Volumetric Adsorption Equilibrium

Prior to evaluating the objective function given in eq S2, we preprocess the experimental volumetric equilibrium data from Section 2.2.2 of the main text as follows:

- We concatenate all the experimental data for a given adsorbent, resulting in a total number of N_{t} data points
- We scale all the isotherms at different temperatures (293.15 K, 303.15 K and 313.15 K) for each adsorbent. For measurements from a specific temperature, every data point is divided by the maximum adsorbed amount at that given temperature, and multiplied by the maximum value of the adsorbed amount for that adsorbent.

S3.2 Simultaneous Estimation of Adsorption Equilibrium and Kinetics

Prior to evaluating the objective function given in eq S2, we preprocess the experimental response from Section 4.2 of the main text and the computational response from Section 5.2 of the main text as follows:

- We downsample the experimental or computational response with a significantly longer duration when compared to the ones with the shortest duration to ensure that the objective function is not dominated by the longer responses
- We concatenate all the experimental or computational responses considered for the parameter estimation, resulting in a total number of N_{t} data points
- We scale the experimental responses from Section 4.2 of the main text and computational responses from Section 5.2 of the main text so as to have comparable magnitudes (each composition is subtracted by the minimum composition obtained from the concatenated data and divided by the maximum difference of the composition and the minimum composition obtained from the concatenated data)
- We scale the experimental responses from Section S5.4 so as to have comparable magnitudes at high compositions and low compositions of the responses (divide the compositions into two groups at the median of the concatenated data and scale the individual groups using the methodology presented in the previous point) and subsequently minimize the sum of the objective functions obtained from eq S2 for the two groups

S4 Maximum Likelihood Estimation and Uncertainty Calculation

Based on certain underlying assumptions described elsewhere, we can show that for maximum likelihood estimation (MLE),[5] the parameter vector $\boldsymbol{\theta}$ that maximizes the probability of observing a given experimental data set, is the minimizer of the function

$$J(\boldsymbol{\theta}) = \frac{N_\mathrm{t}}{2} \ln \left(\sum_{k=1}^{N_\mathrm{t}} \left(\hat{y}_k - y_k(\boldsymbol{\theta}) \right)^2 \right) \tag{S2}$$

where N_t is the total number of experimentally determined data points, $\hat{y}_k$ is the experimentally determined value of the dependent variable, and $y_k(\boldsymbol{\theta})$ is the corresponding model prediction, for a model parameter vector $\boldsymbol{\theta}$.

We rate the quality of the local minimizer $\boldsymbol{\theta^*}$ using two approaches. First, we visually inspect the fit between the experimental responses and the model predictions. Second, we compute the confidence regions to assess how well a minimizer $\boldsymbol{\theta^*}$ was determined. To this aim, we estimate the positive semidefinite parameter covariance matrix V_θ after approximating the measurement error covariance matrix and computing the sensitivities of the model predictions with respect to the parameters.[5,6] Subsequently, we can obtain the hyperellipsoidal confidence region in the N_p-dimensional parameter space as follows

$$\left\{ \boldsymbol{\theta} \in \mathbb{R}^{N_\mathrm{p}} \mid \left(\boldsymbol{\theta} - \boldsymbol{\theta}^* \right)^\mathrm{T} \boldsymbol{V_\theta}^{-1} \left(\boldsymbol{\theta} - \boldsymbol{\theta}^* \right) \leq F_{\chi^2}^{-1} \left(\eta, N_\mathrm{p} \right) \right\} \tag{S3}$$

where $F_{\chi^2}^{-1}(\eta, N_\mathrm{p})$ is the inverse of the chi-squared cumulative distribution function with N_p degrees of freedom at the probability η. Finally, we take the bounding box of the hyperellipsoidal confidence region and we report the confidence intervals for each parameter.

To visualize the confidence regions in Section 4 of the main text, we sample 1000 set of isotherm parameters from the confidence intervals for each material using a latin hypercube sampling technique (*LHS* function of the `SMT` package in Python 3.8.5[7]). Upon evaluating the amount of gas adsorbed at a given temperature and partial pressure for the 1000 sets of isotherm parameters, using eq 2 of the main text, we find the corresponding minimum and maximum. For all the results presented in this work, the confidence region for a given material at a given temperature corresponds to the area bounded between the minimum and maximum amount of gas adsorbed over the entire partial pressure.

S5 Characterization of Blank Volume

S5.1 Blank Volume Experiments

We performed dedicated experiments for the purpose of characterizing the blank volume of the experimental setup. In all these experiments, we removed the adsorbent, saturated the setup with pure CO_2, and finally switched to pure inert gas (helium) using a switching valve. As explained in Section 3.1.1 of the main text, we have divided the entire blank volume of the setup into two segments. To characterize these segments, we performed two distinct sets of experiments. To characterize *Segment I*, we used the same setup configuration as shown in Figure 1 of the main text, and we performed the experiments without the adsorbent. To characterize *Segment II*, we removed all the components of *Segment I* and fed the gas directly to the tee connector that splits the gas into two streams, one to the MS and the other to the vent. To elucidate the impact of flow rate on the blank response, we performed

these experiments at four different flow rates. We performed experiments to characterize *Segment I* at a gas flow rate $F^{\text{in}} = [0.2\ 0.3\ 0.5\ 1.1]^{\text{T}}\ \text{cm}^3\,\text{s}^{-1}$ and to characterize *Segment II* at a gas flow rate $F^{\text{in}} = [0.2\ 0.3\ 0.8\ 1.1]^{\text{T}}\ \text{cm}^3\,\text{s}^{-1}$ at room temperature and pressure.

S5.2 Blank Volume Model

Here, we formulate a generic mathematical model for the blank volume (*Segment I* and *Segment II*) in the experimental setup shown in Figure 1 of the main text (blue and green) by modeling individual components of the setup that are lumped into sections that exhibit a given flow behavior.[8–11] To elucidate the flow behavior, we conducted a few preliminary blank experiments based on the approach described in Section S5.1. The responses obtained from these experiments exhibited either an exponential decay response (see Figure S4a) or a double exponential decay response (see Figure S4b,c). The former response is characteristic of a plug flow behavior with axial dispersion. The latter response is characteristic of two volumes in series, namely one that exhibits a plug flow behavior with axial dispersion (for the time shift) and the other that exhibits a diffusive mixing behavior (for the double exponential). We have modeled these two segments by drawing inspiration from compartment models used in chemical reaction engineering,[12] that facilitates describing plug flow, mixing, diffusion and stagnation behavior of fluids. A schematic that visualizes the volumes in the two segments as a combination of chemical reactors is shown in Figure 1 of the main text. Note that the model equations presented in this section are written for the adsorbing gas in a binary gas mixture.

Segment I: The entire segment, shown in blue in Figure 1 of the main text, has the same gas flow rate $F(t)$ as the outlet of the CSTR that models the gas adsorption in the adsorbent. We described the gas flow in the volume that exhibits a plug flow behavior with axial dispersion using a series of N^{S} CSTRs, with a total volume V^{S} [m^3]. Note that the higher the number of CSTRs N^{S}, the lower the axial dispersion. The mass balance around a CSTR p is given as

$$\frac{\mathrm{d}c_p^{\text{S}}}{\mathrm{d}t} = \frac{F(t)}{V^{\text{S}}/N^{\text{S}}}\left(c_{p-1}^{\text{S}} - c_p^{\text{S}}\right) \qquad p = 1, \dots, N^{\text{S}} \tag{S4}$$

where c_p^{S} [mol m^{-3}] is the concentration of the gas at the outlet of CSTR p and $F(t)$ is the volumetric mixture gas flow rate at the outlet of the CSTR that models the gas sorption in the adsorbent, obtained by solving the system of equations given by eq 8 of the main text.

We described the gas flow in the volume that exhibits a diffusive mixing behavior as two CSTRs in parallel, characterized by a mixing volume V^{M} [m^3] with a volumetric mixture gas flow rate $\left(F(t) - F^{\text{D}}\right)$ [m^3 s^{-1}] and a diffusive volume V^{D} [m^3] with a constant volumetric mixture gas flow rate F^{D} [m^3 s^{-1}]. The mass balance around these two reactors is given by

$$\begin{aligned} \frac{\mathrm{d}c^{\text{M}}}{\mathrm{d}t} &= \frac{\left(F(t) - F^{\text{D}}\right)}{V^{\text{M}}}\left(c_{N^{\text{S}}}^{\text{S}} - c^{\text{M}}\right) \\ \frac{\mathrm{d}c^{\text{D}}}{\mathrm{d}t} &= \frac{F^{\text{D}}}{V^{\text{D}}}\left(c_{N^{\text{S}}}^{\text{S}} - c^{\text{D}}\right) \end{aligned} \tag{S5}$$

where c^{M} [mol m^{-3}] and c^{D} [mol m^{-3}] are the concentrations of the gas at outlet of the mixing and diffusive volumes, respectively, and $c_{N^{\text{S}}}^{\text{S}}$ [mol m^{-3}] is the concentration at the outlet of CSTR N^{S}, obtained from eq S4.

Finally, we combine the gas from the mixing and the diffusive volume, which is the outlet from *Segment I*, with a gas concentration c^{S} [$\mathrm{mol\,m^{-3}}$] defined as

$$c^{\mathrm{S}} = \frac{\left(F(t) - F^{\mathrm{D}}\right) c^{\mathrm{M}} + F^{\mathrm{D}} c^{\mathrm{D}}}{F(t)} \tag{S6}$$

where c^{M} and c^{D} are obtained from eq S5. Note that we assumed the pressure and temperature of the gas from the two volumes to be identical. The initial conditions for eqs S4 and S5 are

$$\begin{aligned} c_p^{\mathrm{S}}(0) &= \frac{Py^0}{RT} \qquad p = 1, \ldots, N^{\mathrm{S}} \\ c^{\mathrm{M}}(0) &= \frac{Py^0}{RT} \\ c^{\mathrm{D}}(0) &= \frac{Py^0}{RT} \end{aligned} \tag{S7}$$

where P [Pa] is the pressure, T [K] is the temperature and y^0 [–] is the initial gas phase mole fraction in the blank volume of the setup.

Segment II: The entire segment, shown in green in Figure 1 of the main text, has a gas flow rate of F^{MS} [$\mathrm{m^3\,s^{-1}}$] and it is dictated by the vacuum pump of the MS. We described the gas flow using a series of N^{MS} CSTRs, with a total volume V^{MS} [$\mathrm{m^3}$]. The mass balance around a CSTR q is given as

$$\frac{\mathrm{d}c_q^{\mathrm{MS}}}{\mathrm{d}t} = \frac{F^{\mathrm{MS}}}{V^{\mathrm{MS}}/N^{\mathrm{MS}}} \left(c_{q-1}^{\mathrm{MS}} - c_q^{\mathrm{MS}}\right) \qquad q = 1, \ldots, N^{\mathrm{MS}} \tag{S8}$$

where c_q^{MS} [$\mathrm{mol\,m^{-3}}$] is concentration of the gas at outlet of CSTR q and F^{MS} [$\mathrm{m^3\,s^{-1}}$] is a constant volumetric mixture gas flow rate through the MS, which is independent of the volumetric mixture gas flow rate $F(t)$ in *Segment I*. The initial condition for eq S8 is

$$c_q^{\mathrm{MS}}(0) = \frac{Py^0}{RT} \qquad q = 1, \ldots, N^{\mathrm{MS}} \tag{S9}$$

where P [Pa] is the pressure, T [K] is the temperature and y^0 [–] is the initial gas phase mole fraction in the blank volume of the MS.

S5.3 Estimating Blank Volume Model Parameters

We obtained the blank volume model parameters by following a two-step approach. First, we obtain the model parameters for *Segment II*, i.e., for the segment with the MS, by using the corresponding model embedded into a parameter estimator. Second, using the estimated model parameters for *Segment II*, we obtain the model parameters for *Segment I*, i.e., for the segment with the adsorption cell, the MFM, and the gas lines, by using a combined model comprised of *Segment I* and *Segment II* embedded into a parameter estimator.

Prior to performing parameter estimation, we preprocessed the raw experimental data, obtained using the protocol described in Section S5.1, through the steps outlined in Section S3. We obtained the minimizer θ^*, using a maximum likelihood estimator described in Section S4, for the models corresponding to the blank volume. We solved the minimization problem, given by eq S2, to estimate the model parameters using an evolutionary optimization algorithm, i.e., a standard and elitist genetic algorithm (`geneticalgorithm2`[46] (v6.2.4) in Python 3.8.5). In eq S2, for all the cases, $\hat{y}_k$ is defined as

the kth point of the experimentally measured outlet CO_2 gas composition obtained from the MS and $y_k^{MS}(\boldsymbol{\theta})$ is defined as the corresponding model prediction for a given model parameter vector $\boldsymbol{\theta}$. For the blank volume model of *Segment I*, the model parameter vector $\boldsymbol{\theta} = \left[N^S \; V^S \; F^D \; V^M \; V^D\right]$ and for the blank volume model of *Segment II*, the model parameter vector $\boldsymbol{\theta} = \left[N^{MS} \; V^{MS}\right]$.

As detailed in Section 3.3.2 of the main text, to ensure we move toward the true minimizer, we have structured each parameter estimation run to consist of five local iterations. We executed each of these local iterations for a total of 30 generations with a population size of 200. The initial population used for each of the local iteration comes from the final population of the previous local iteration (except for the first iteration). The model parameter vector at the end of the fifth iteration is assumed to be the minimizer $\boldsymbol{\theta}^*$. The upper and lower bounds used for each of the parameters, along with their corresponding type (discrete or continuous), is provided in Table S2.

S5.4 Characterization of Blank Volume

We performed several experiments, using the protocol described in Section S5.1, to characterize the blank volume in the two segments (*Segment I* and *Segment II*) of the experimental setup. When characterizing *Segment I* of the experimental setup, we considered two sub-cases. These two sub-cases correspond to configurations with (referred to as *Segment I w/ Ball*, applied for AC and 13X) or without (referred to as *Segment I w/o ball*, applied for BN) the stainless steel ball placed inside the adsorption cell to reduce the blank volume and to increase the heat dissipation. Subsequently, we estimated the blank volume model parameters for the corresponding experimental configuration using the methodology presented in Section S5.3.

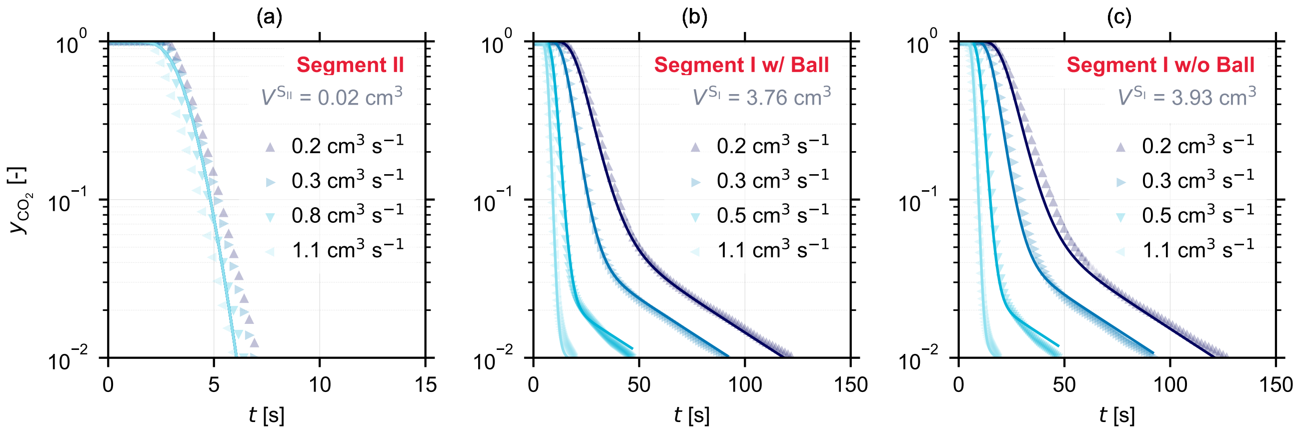

Figure S4: Experimental and simulated blank responses for **(a)** *Segment II*, *Segment I* **(b)** with a stainless steel ball and **(c)** without a stainless steel ball in the adsorption cell. The corresponding blank volume for *Segment I* and *Segment II* is provided in each panel. The markers represent the time evolution of the experimental CO_2 composition y_{CO_2} at four different flow rates. The solid curves indicate the corresponding simulated response generated using the model described in Section S5.2 with model parameters given in Table S1. The stainless steel ball has been used in some experiments to reduce the blank volume of the setup and to help dissipate heat generated during gas adsorption.

The time-resolved experimental and the simulated blank responses y_{CO_2} for *Segment I* (both w/o Ball and w/ Ball) and *Segment II* are shown in Figure S4. The estimated blank volume model parameters are provided in Table S1. We can make three observations based on the experimental and

the simulated outcomes. First, we can observe differences in the experimental response (markers) obtained from Segment I and Segment II. The response from the former, shown in Figure S4b,c, is flow dependent and is characteristic of volumes that exhibit a plug flow behavior (for the time shift) coupled with a diffusive mixing behavior (for the double exponential). The response from the latter, shown in Figure S4a, is flow independent and is characteristic of plug flow behavior with axial dispersion.[12] Second, in all the three cases, the simulated blank response (solid lines) agrees well with the experimental response, indicating a good choice of mathematical model to describe the blank volumes in the experimental setup. We can attribute the minor deviations between the experiments and simulated model responses to errors arising from gas composition estimation from the MS (see Section S2.4) and from the parameter estimator. Finally, as expected, the absolute estimated blank volume of *Segment II* $V^{\mathrm{S_{II}}}$ is the lowest (and very small) and of the *Segment I* without the ball (*Segment I w/o ball*) $V^{\mathrm{S_I}}$ is the highest.

Even though not highlighted explicitly and not evident upon reading the preceding discussion, the accurate characterization of the blank volume of the experimental setup has implications on the predictions of the adsorption equilibria and kinetics when using small quantities of adsorbents. To highlight this, we have performed a thorough analysis on the impact of inaccurate blank volume models on the predicted adsorption behavior and this analysis is discussed in detail in Section S7.3.

S6 Estimation of Equilibrium and Kinetics: Experimental

S6.1 Repeatability of Experiments

We carried out dynamic sorption experiments on the three materials using the protocol described in Section 3.1.2 of the main text. To check repeatability of experiments, we carried out experiments in ascending order of temperature over the course of 6 h. Subsequently, we degassed the adsorbent – *in situ* – overnight using the protocol described in Section S2.2, and we repeated the experiments in descending order of temperature. For the case of 13X, we followed a different degassing protocol. We degassed the adsorbent – *in situ* – overnight between experiments conducted at every single temperature to minimize the effect of water adsorption on the experimental responses.

The repetitions of the experimental desorption responses for all the three materials are illustrated in Figure S5. We can observe that all but one experiment, performed at three different temperatures and two different flow rates, are repeatable. We can observe that the experiment performed at 326 K on 13X is not repeatable. We can attribute this to the different degassing temperature used prior to the experiments. For one repetition we used a degassing temperature of 423 K, while for the other, we used 473 K. This might have led to trace amounts of moisture remaining in the adsorbent, degassed at a lower temperature, prior to the start of the experiment.

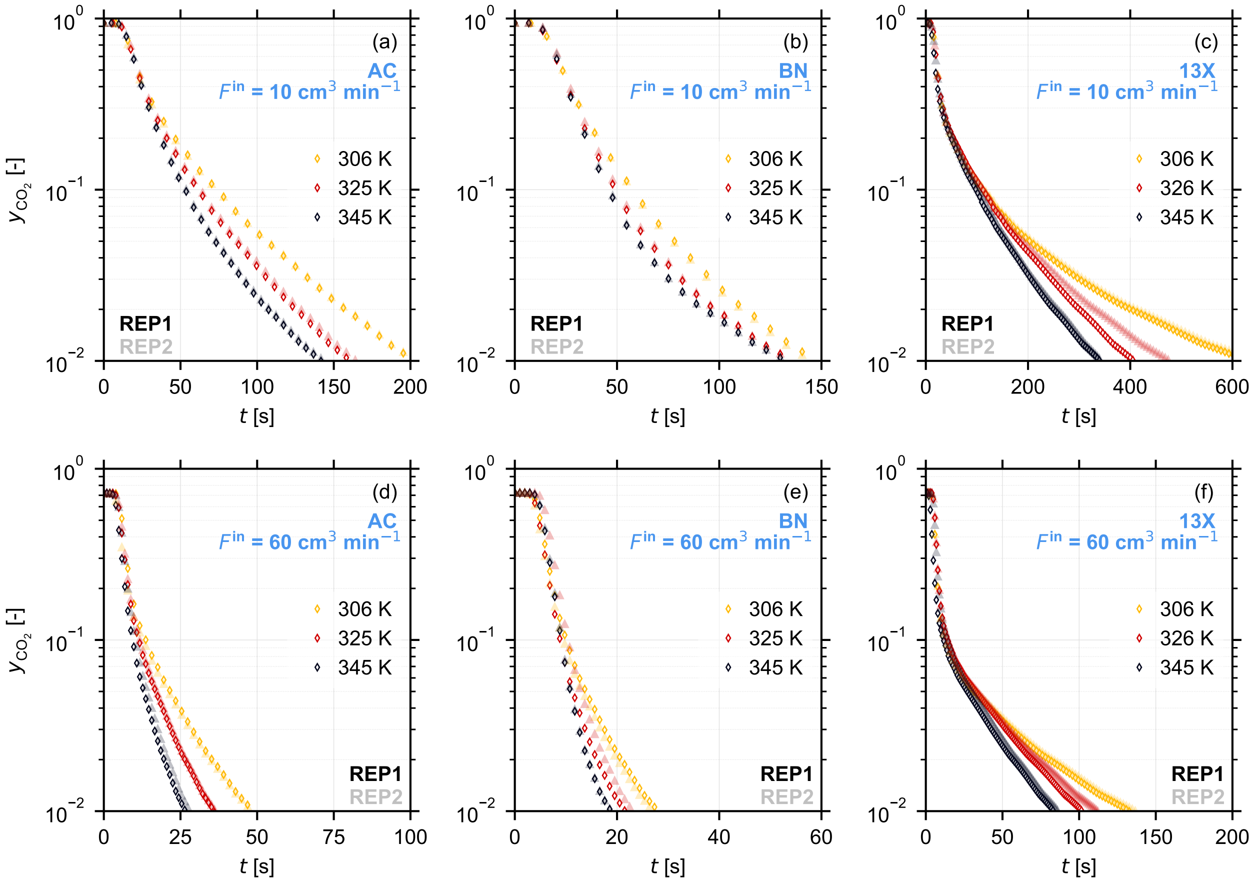

Figure S5: Repetitions of experimental desorption responses at 306 K (yellow), 325 K (red), and 345 K (black), $F^{in} = 10\,cm^3\,min^{-1}$ and $y_0 = 0.94$ for **(a)** Norit RB3 (AC), **(b)** boron nitride (BN) and **(c)** Zeolite 13X (13X) using the approach presented in Section 3.1 of the main text. The repetitions of experimental desorption responses for the three materials at 306 K (yellow), 325 K (red), and 345 K (black), $F^{in} = 60\,cm^3\,min^{-1}$ and $y_0 = 0.73$ are shown in panels **(d)** through **(f)**. The markers represent the time evolution of the experimental CO_2 composition y. The two repetitions of the experiments at each condition is distinguished by the different shades (darker shade: REP1 and lighter shade: REP2) of the marker color.

S6.2 Flow Dependency on Adsorption Behavior

To enable accurate estimation of both adsorption equilibria and kinetics from desorption responses, one should perform experiments at different inert gas flow rates. Low flow rate experiments enable accessing the equilibrium regime, while high flow rate experiments enable accessing the kinetic regime of adsorption. We can check the regimes being accessed by a given flow rate on a given material by visualizing the desorption response in the so-called Ft plot,[13] shown in Figure S6. In a typical Ft plot, if the curves overlap at different flow rates, we cannot extract any kinetic information as the adsorbent-gas system is under equilibrium. In Figure S6, due to the presence of blank volumes in the system, we cannot directly plot the curves at different flow rates. Therefore, once we have estimated the adsorption equilibria and kinetic parameters, we feed them into the gas adsorption model described in *Gas Adsorption in the Adsorbent* in Section 3.2 of the main text. Subsequently, we plot the desorption responses at the outlet of the adsorption cell. We can observe that for all the three materials, the Ft curves at the two different flow rates explored in this work do not overlap. Therefore, we can be confident

that both the equilibrium and kinetic parameters are satisfactorily extracted and well-determined.

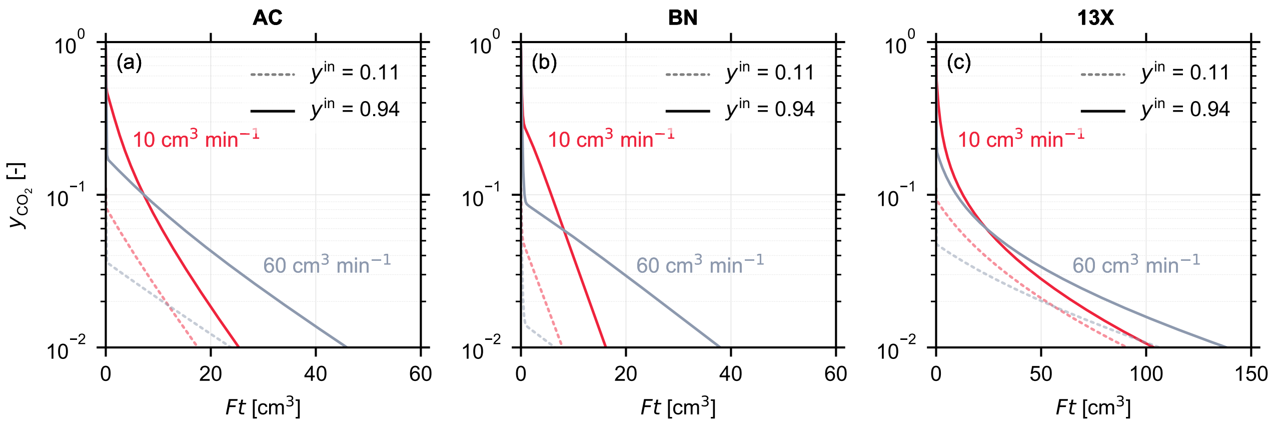

Figure S6: Time-resolved evolution of CO_2 composition as a function of the product of outlet gas flow rate and time Ft for **(a)** Norit RB3 (AC), **(b)** boron nitride (BN), and **(c)** Zeolite 13X (13X) pellets at 308.15 K, $F^{\text{in}} = 10\,\text{cm}^3\,\text{min}^{-1}$ (red) $y_0 = 0.12$ (dashed) and$y_0 = 0.94$ (solid); and $F^{\text{in}} = 60\,\text{cm}^3\,\text{min}^{-1}$ (gray) $y_0 = 0.11$ (dashed) and $y_0 = 0.73$ (solid). The curves were obtained using the gas adsorption model described in *Gas Adsorption in the Adsorbent* in Section 3.2 of the main text with the parameters given in Table 2 of the main text.

S6.3 Objective Function

To understand the robustness of the optimization technique used in this work, for all the three materials we repeated the parameter estimation five times, using the methodology discussed in Section 3.3.2 of the main text. The objective function values $J(\theta)$, given by eq S2, obtained at the local minimizer $\boldsymbol{\theta}^*$ for the five repeats of the parameter estimation for all the three materials with the experimental and the digital twin response is provided in Figure S7. For the experimental responses, obtaining the actual value of the objective function with the true equilibria and the kinetics of a given material is not possible. However, for the digital twin responses, we can compare the objective function values $J(\theta)$ obtained through the five parameter estimation repeats with the *true* objective function J_{true}. The latter can be obtained by evaluating the objective function, given by eq S2, with adsorption equilibrium and kinetic parameters, reported in Table S3, used to generate the computational responses. We can make two observations from Figure S7. First, the differences in the objective function values for the different repeats of a given material is very small. Second, for the computational case, we obtain higher values of objective function when compared to the *true* objective function J_{true}. This leads to a situation in which the optimizer is unable to reach the true minima of the problem, and therefore prevents from obtaining the true adsorption equilibrium and kinetic parameters.

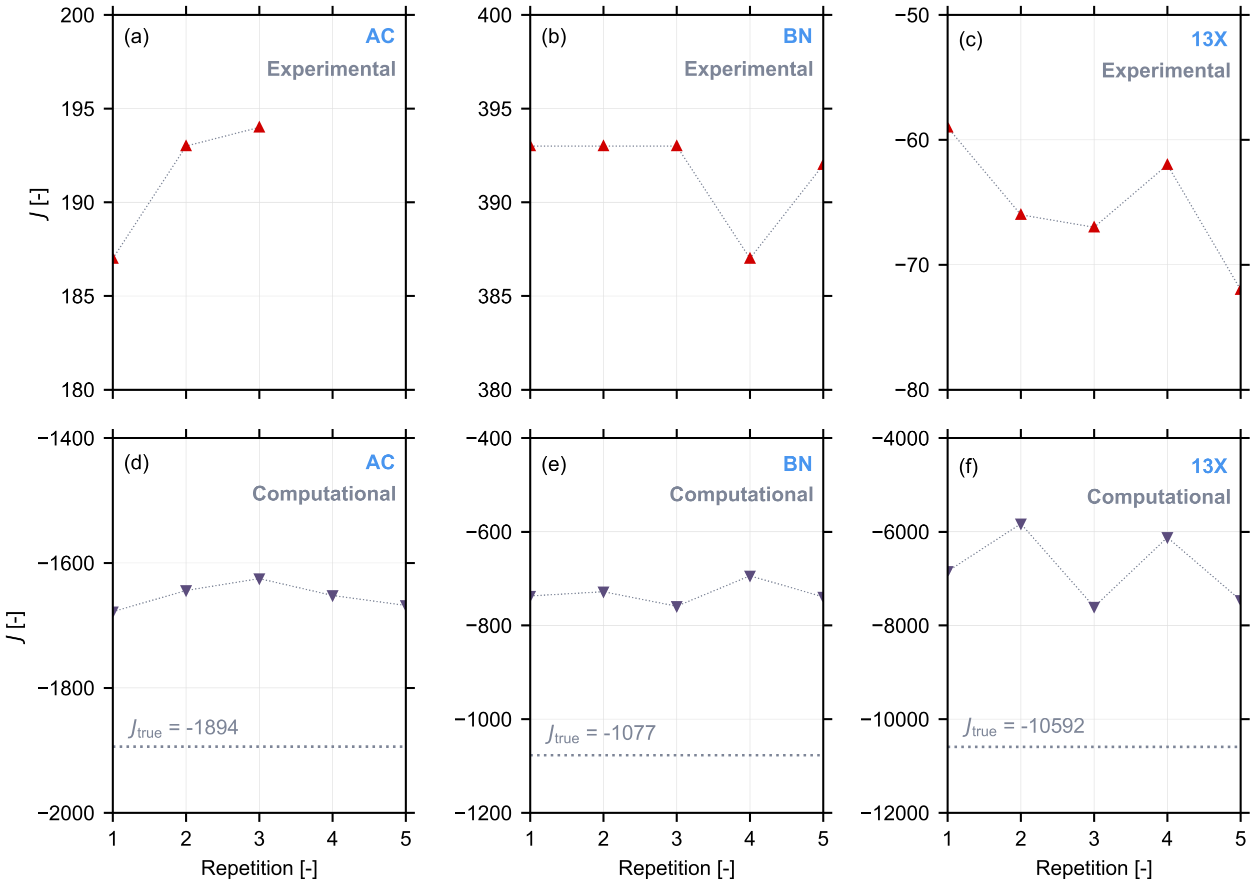

Figure S7: Objective function values J at the end of each repetition obtained by performing the parameter estimation routine to estimate equilibrium and kinetic parameters, using the approach described in Section 3.3.2 of the main text, with the experimental responses for **(a)** Norit RB3 (AC), **(b)** boron nitride (BN) and **(c)** Zeolite 13X (13X). The corresponding objective function value J at the end of each repetition with the response obtained from the digital twin described in Section 5.2 of the main text are shown in panels **(d)** through **(f)**. In panels (d) through (f), the objective function obtained by using the equilibrium and kinetic parameters of the digital twin given in Table S3 is also additionally provided (J_{true}).

S6.4 Model Parameter Values and Bounds

Table S1: Parameters for the blank volume models, described in Section S5.2 and shown in Figure S4, of the mass spectrometer (MS) and the experimental setup. The blank volume for the experimental setup was characterized in two configurations: one with and one without a stainless steel ball in the adsorption column.

Parameter	Unit	Value
**MS**		
N^{MS}	-	16
V^{MS}	cm^3	0.02
**Setup w/ ball**		
N^{S}	-	15
V^{S}	cm^3	2.67
F^{D}	$\mathrm{cm}^3\,\mathrm{s}^{-1}$	0.01
V^{M}	cm^3	1.09
V^{D}	cm^3	0.07
**Setup w/o ball**		
N^{S}	-	15
V^{S}	cm^3	2.85
F^{D}	$\mathrm{cm}^3\,\mathrm{s}^{-1}$	0.01
V^{M}	cm^3	1.08
V^{D}	cm^3	0.07

Table S2: Parameter bounds and variable types used in the parameter estimation routine, described in Section S5.3 and Section 3.3.2 of the main text, respectively, for the characterization of the blank volume of the experimental setup and of the equilibrium and kinetics of CO_2 on the three materials used in this work.

Parameter	Unit	Bounds		Variable Type
		Low	High	
**Isotherm Parameters**				
q_{sb}	$\mathrm{mol\,kg^{-1}}$	0	10	Continuous
b_0	$(\times 10^{-7})\ \mathrm{m^3\,mol^{-1}}$	0	100	Continuous
$-\Delta U_{\mathrm{b}}$	$\mathrm{kJ\,mol^{-1}}$	0	40	Continuous
q_{sd}	$\mathrm{mol\,kg^{-1}}$	0	10	Continuous
d_0	$(\times 10^{-7})\ \mathrm{m^3\,mol^{-1}}$	0	100	Continuous
$-\Delta U_{\mathrm{d}}$	$\mathrm{kJ\,mol^{-1}}$	0	40	Continuous
**Kinetic Parameters**				
k_1	$\mathrm{s^{-1}}$	0	1000	Continuous
k_2	$\mathrm{s^{-1}}$	0	1000	Continuous
**Segment I Blank Volume Model Parameters**				
N^{S}	-	1	30	Discrete
V^{S}	$\mathrm{cm^3}$	0	10	Continuous
F^{D}	$\mathrm{cm^3\,min^{-1}}$	0	0.05	Continuous
V^{M}	$\mathrm{cm^3}$	0	10	Continuous
V^{D}	$\mathrm{cm^3}$	0	10	Continuous
**Segment II Blank Volume Parameters**				
N^{MS}	-	1	30	Discrete
V^{MS}	$\mathrm{cm^3}$	0	10	Continuous

S7 Estimation of Equilibrium and Kinetics: Computational

S7.1 Model Parameter Values

Table S3: Parameter values for the equilibrium and kinetics and for other variables used in the digital twin, described in Section 5.2 of the main text, for Norit RB3 (AC), boron nitride (BN) and Zeolite 13X (13X). The flow rates, temperatures, and initial CO_2 composition used to generate the computational responses are also provided.

Parameter	Unit	Value		
		**AC**	**BN**	**13X**
**Isotherm Parameters**				
q_{sb}	$\mathrm{mol\,kg^{-1}}$	0.47	7.01	3.83
b_0	$(\times 10^{-7})\ \mathrm{m^3\,mol^{-1}}$	102.00	2.32	0.13
$-\Delta U_{\mathrm{b}}$	$\mathrm{kJ\,mol^{-1}}$	25.10	24.90	40.00
q_{sd}	$\mathrm{mol\,kg^{-1}}$	6.51	-	2.57
d_0	$(\times 10^{-7})\ \mathrm{m^3\,mol^{-1}}$	3.51	-	48.80
$-\Delta U_{\mathrm{d}}$	$\mathrm{kJ\,mol^{-1}}$	25.70	-	35.16
**Kinetic Parameters**				
k_1	$\mathrm{s^{-1}}$	1.02	0.08	664.00
k_2	$\mathrm{s^{-1}}$	16.79	302.96	76.10
**Other Variables**				
ϵ_{T}	-	0.61	0.88	0.79
ρ_{s}	$\mathrm{g\,cm^{-3}}$	1.68	3.40	4.10
m_{ads}	mg	62.5	79.7	79.0
**Simulated Conditions**				
T	K	$[308.15\ 328.15\ 348.15]^{\mathrm{T}}$		
F^{in}	$\mathrm{cm^3\,min^{-1}}$	$[10.0\ 60.0]^{\mathrm{T}}$		
y_0	-	$10.0\,\mathrm{cm^3\,min^{-1}}$: $[0.12\ 0.94]^{\mathrm{T}}$<br>$60.0\,\mathrm{cm^3\,min^{-1}}$: $[0.11\ 0.73]^{\mathrm{T}}$		

S7.2 Computationally Generated and Fitted Desorption Responses

The computationally generated time-resolved CO_2 gas composition y_{CO_2} for the 12 *in silico* experiments performed at four initial gas phase composition, two inert gas flow rates, and three temperatures (using the digital twin described in Section 5.2 of the main text), along with the corresponding model fit, with the lowest objective function, for all the materials is provided in Figure S8. We can make several observations based on both the *in silico* experimental and the simulation outcome that are common for all three materials. First, similar to the experimental studies shown in Section 4.2 of the main text, as expected, the *in silico* experiment performed at lower temperature takes longer to reach a given gas phase composition at a given flow rate than an experiment performed at higher temperature, indicating

a higher adsorption capacity at a lower temperature. Second, we can observe a significant time lag between the blank response (light gray) and the desorption response with the adsorbent. Finally, we can see that the overall fit of the simulated response is in good agreement with the *in silico* experimental response for all the three materials throughout the entire composition range. Note that we can observe minor deviations for some flow and temperature conditions in all the three materials. These minor deviations lead to the observed discrepancies in the estimated isotherms when compared to the TRUE isotherms, shown in Figure 6 of the main text.

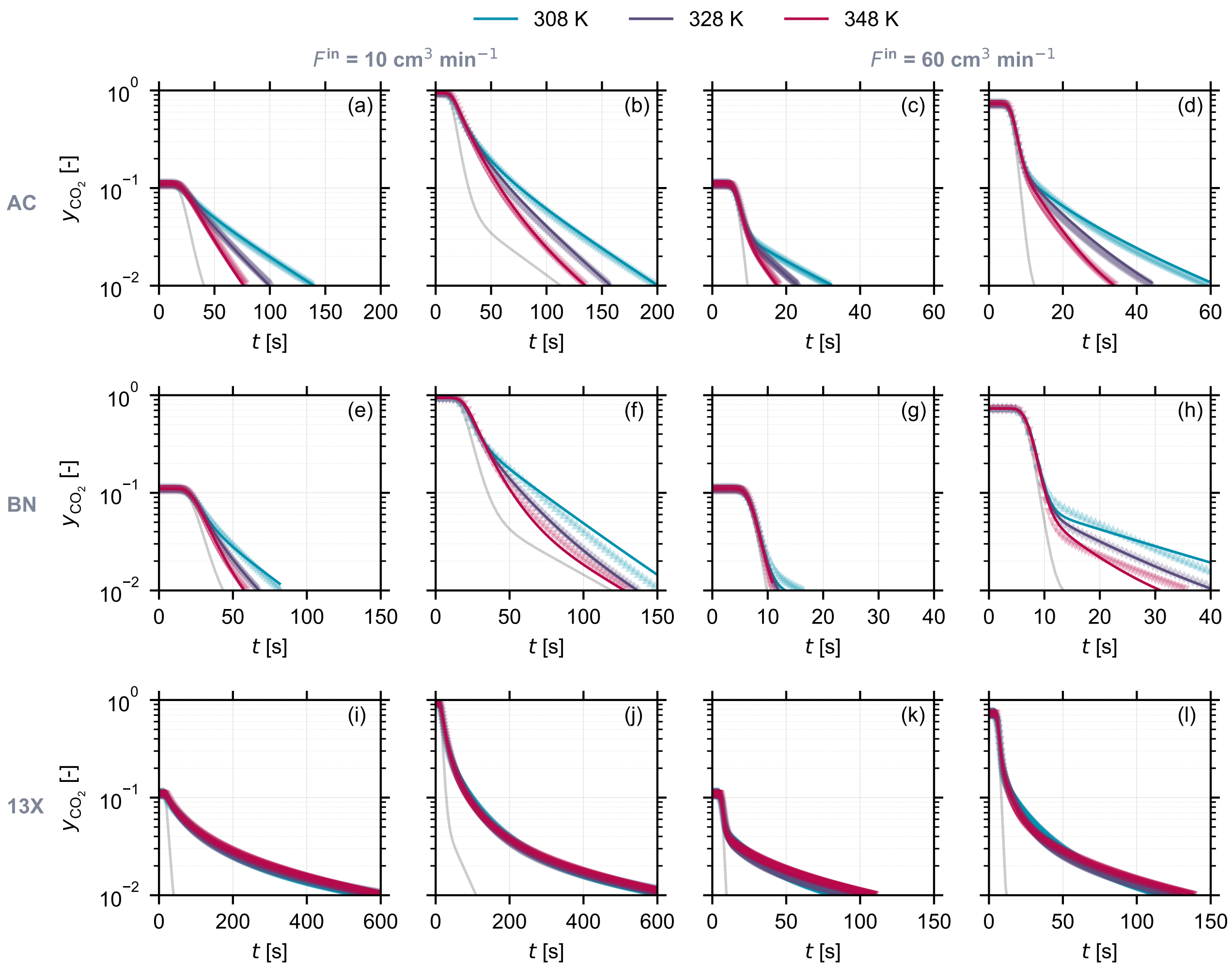

Figure S8: *In silico* experimental and simulated desorption responses for Norit RB3 (AC, a–d), boron nitride (BN, e–h), and Zeolite 13X (13X, i–l) pellets at 308.15 K (teal), 328.15 K (blue), and 348.15 K (rose), $F^{\text{in}} = 10\,\text{cm}^3\,\text{min}^{-1}$ **(a, e, i)** $y_0 = 0.12$ and **(b, f, j)** $y_0 = 0.94$; and $F^{\text{in}} = 60\,\text{cm}^3\,\text{min}^{-1}$ **(c, g, k)** $y_0 = 0.11$ and **(d, h, l)** $y_0 = 0.73$. The markers represent the time evolution of the CO_2 composition y obtained using the digital twin described in Section 5.2 of the main text using the parameters given in Table S3. The solid curves indicate the corresponding simulated response generated using the model described in Section 3.2 of the main text with the parameters corresponding to the parameter estimation repeat with the lowest objective function. The gray line in each panel represents the blank response of the setup at the given flow rate and initial gas phase composition.

S7.3 Sensitivity to Model Inputs

In this second study to evaluate the accuracy of the proposed methodology, we want to understand the sensitivity of the model inputs, e.g., porosity, blank volume model, to accurately estimate the adsorption equilibria and kinetics of the materials. To this aim, we perform a full curve fit of the computationally generated responses for AC, using the simulation framework described in Section 3.3 of the main text. However, we provide model inputs that are different from the *true* parameter values used to generate the response. For example, to understand the sensitivity of the porosity ϵ_T on the accuracy of the estimates of the adsorption behavior, we generate the computational responses with the digital twin using a porosity $\epsilon_\mathrm{T} = 0.61$, but we use a porosity of either $\epsilon_\mathrm{T} = 0.35$ or $\epsilon_\mathrm{T} = 0.90$ (see Figure S9) for the parameter estimation routine. The former is analogous to obtaining an experimental response from a material that exhibits a given porosity, and the latter is analogous to an incorrect experimental characterization of the porosity of the material and subsequently using it for the full curve fit in the simulation framework described in Section 3.3 of the main text.

Here, we have performed a sensitivity analysis on the impact of the porosity, mass of the adsorbent, and the blank volume model on the accuracy of the estimated adsorption behavior, specifically on the adsorption isotherm. For the impact of porosity, we examine the isotherms obtained by using the aforementioned values, i.e., $\epsilon_\mathrm{T} = 0.35$ or $\epsilon_\mathrm{T} = 0.90$ for the parameter estimation routine with $\epsilon_\mathrm{T} = 0.61$ as the reference used to generate the computational responses. For the impact of the mass of adsorbent, we examine the isotherms obtained by using $m_\mathrm{ads} = 59.38\,\mathrm{mg}$ or $m_\mathrm{ads} = 65.63\,\mathrm{mg}$ for the parameter estimation routine with $m_\mathrm{ads} = 62.50\,\mathrm{mg}$ as the reference used to generate the computational responses. This is analogous to an incorrect estimation of the mass of the adsorbent between *ex situ* and *in situ* degassing, described in Section S2.2, arising from adsorption of atmospheric moisture. Even though in a real experiment, water adsorption will not have an impact on the actual adsorption behavior due to *in situ* degassing, it might have an impact on the analysis of the experimental responses due to an incorrect mass input to the model during the parameter estimation stage. Finally, for the impact of the blank volume model, we examine the isotherms obtained by using a model that assumes the entire blank volume (*Segment I* and *Segment II*) to exhibit either a plug flow behavior with axial dispersion (tanks in series, TIS) or a combination of a plug flow behavior with axial dispersion and a diffusive mixing behavior (TIS + D/M). As a reference to generate the computational response, we have used a combination of a plug flow behavior with axial dispersion and a diffusive mixing behavior to describe the blank volume in *Segment I* and a plug flow behavior with axial dispersion to describe the blank volume in *Segment II* (TIS + D/M + MS). Unlike the case of porosity or mass of the adsorbent, to use different blank volume models in the parameter estimation stage, we reevaluated the corresponding model parameters by refitting the experimental response shown in Figure S4, using the TIS and the TIS + D/M model. The experimental and model responses for all the three models are visualized in Figure S10. We subsequently used the corresponding blank volume model parameters as an input for the parameter estimation routine.

The CO_2 isotherms for AC at three different temperatures as a function of its partial pressure $q^*_{CO_2}(P, T)$, obtained from the full curve fit and the aforementioned model inputs, are shown in Figure S9. We can make several key observations. First, for all the three cases, i.e., porosity (panel (a)), mass (panel (b)), and blank volume model (panel (c)), the choice of the parameter or model values has an impact on the estimated isotherms. Second, an overestimation of the porosity ($\epsilon_\mathrm{T} = 0.90$) has a higher deviation in the adsorption capacity from the reference case, when compared to the underes-

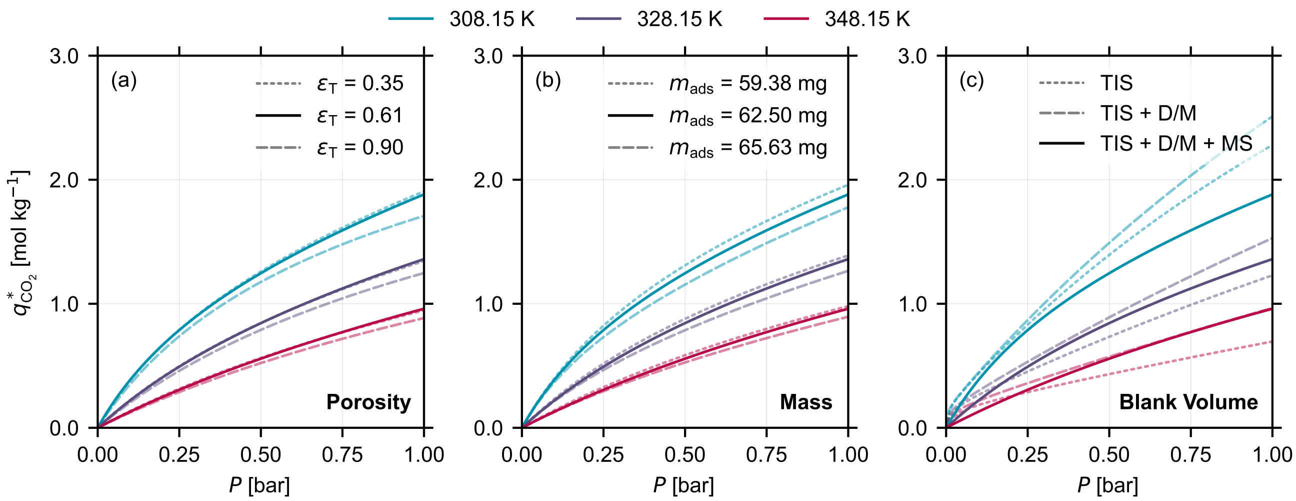

Figure S9: Equilibrium isotherms $q^*(P,T)$ for CO_2 estimated at 308.15 K (teal), 328.15 K (blue), and 348.15 K (rose), using the approach presented in Section S7.3, for Norit RB3 (AC) to study the sensitivity of incorrect **(a)** total porosity, **(b)** mass of the pellet and **(c)** blank volume model. In all the three panels, the solid line corresponds to estimate shown in Figure 6 of the main text with the lowest objective function. In panel (a), the total porosity ϵ_T used in the model is chosen between 0.35 (dotted) and 0.90 (dashed). In panel (b), the mass of the pellet used in the model is assumed to be measured with an error of ±5 % from the actual mass (62.5 mg). In panel (c), three blank volume models are considered to represent the actual blank volume of the system: TIS (dotted, tanks in series for the entire setup), TIS + D/M (dashed, one set of tanks in series and one diffusive and one mixing volume for the entire setup) and TIS + D/M + MS (solid, tanks in series with diffusive and mixing volumes in the blue section of Figure 1 of the main text and tanks in series for the MS in the green section of Figure 1 of the main text, see panel (b) of Figure S4). The blank volume model response for these three cases is shown in Figure S10.

timation of the porosity ($\epsilon_T = 0.35$). We can attribute this to a significantly higher gas phase volume for $\epsilon_T = 0.90$ ($V_g = 9V_s$), given by eq 4 of the main text, for the same volume of solid when compared to $\epsilon_T = 0.61$ ($V_g = 1.6V_s$) and $\epsilon_T = 0.35$ ($V_g = 0.5V_s$). Since the total amount of gas in the system is fixed for a given desorption response, an increase in the volume of the gas phase translates to a reduction of gas in the adsorbent. This leads to a reduction in the estimated adsorption capacity at that condition. Third, an overestimation of the mass of the adsorbent ($m_{ads} = 65.63$ mg), leads to an underestimation of the adsorption capacity and vice versa. Since the total amount of gas in the system is fixed for a given desorption response, an overestimation of the mass of adsorbent translates to the same quantity of gas being adsorbed on a larger quantity of adsorbent when compared to the reference case. This leads to a lower estimated adsorption capacity on a unit mass basis. Finally, an incorrect choice of the blank volume model can lead to errors in the estimated adsorption capacity of around 25 % (see TIS and TIS + D/M at 308.15 K) when compared to the reference case (TIS + D/M + MS). We can attribute this observation to a poor agreement of the blank volume model responses for TIS and TIS + D/M with the experimental response, as shown in Figure S10. In more detail, depending on the flow rate of the gas and the blank volume model chosen, we either underpredict or overpredict the blank volume of the setup. Further, due to mass balance constraints, this will lead to a corresponding overprediction or underprediction of the estimated adsorption capacity, respectively.

To summarize, we can conclude based on the analysis presented here that the efforts we invested in accurately characterizing the textural characteristics of the porous material in Section 4.1 of the main text and the blank volume of the experimental setup in Section S5.4 have a significant impact on accurately characterizing the adsorption behavior. The impact of these quantities is often minimal when using several grams or kilograms of porous material. However, when using small quantities (in the milligram scale) of porous material, like in this work, one should take utmost precaution in ensuring the accurate characterization of the aforementioned quantities.

S7.4 Impact of Blank Volume Model

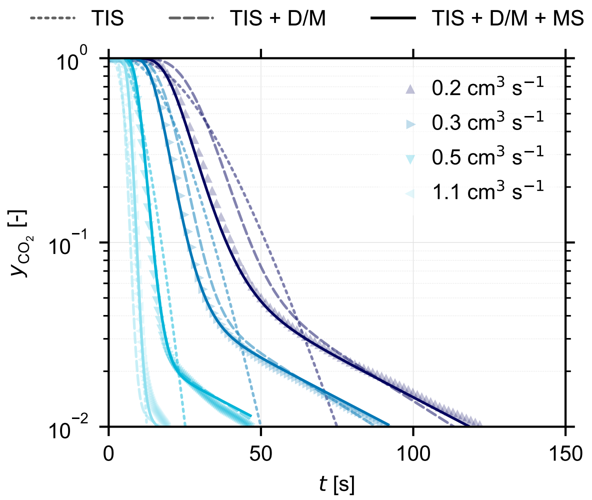

Figure S10: Experimental and simulated blank responses for experimental setup with a stainless steel ball in the adsorption column. The markers represent the time evolution of the experimental CO_2 composition y at four different flow rates. The curves indicate the responses from the three blank volume models considered to represent the actual blank volume of the system: TIS (dotted, tanks in series for the entire setup), TIS + D/M (dashed, one set of tanks in series and one diffusive and one mixing volume for the entire setup) and TIS + D/M + MS (solid, tanks in series with diffusive and mixing volumes in the blue section of Figure 1 of the main text and tanks in series for the MS in the green section of Figure 1 of the main text). The TIS + D/M + MS response is generated using the model described in Section S5.2 with model parameters given in Table S1.

To study the impact of the blank volume model, we have identified three candidate blank volume representations, namely, the entire blank volume (*Segment I* and *Segment II*), described in *Blank Volume* in Section S5.2, to exhibit either a plug flow behavior with axial dispersion (tanks in series, TIS) or a combination of a plug flow behavior with axial dispersion and a diffusive mixing behavior (TIS + D/M). As a reference to generate the computational response, we have used a combination of a plug flow behavior with axial dispersion and a diffusive mixing behavior to describe the blank volume in *Segment I* and a plug flow behavior with axial dispersion to describe the blank volume in *Segment II* (TIS + D/M + MS). We reevaluated the corresponding model parameters by refitting the experimental response, using the aforementioned models. The experimental and model responses for all the three models are visualized in Figure S10. We can observe that the overall fit of the TIS + D/M + MS model is in good agreement with the experimental response for all the flow rates. The other two models, depending on the flow rates, the time required to reach a given composition is either under- or

overpredicted. This will lead to a corresponding under- or overprediction of the blank volume in the experimental setup.

References

[1] Rouquerol, F.; Rouquerol, J.; Sing, K. S. W.; Llewellyn, P.; Maurin, G. *Adsorption by Powders and Porous Solids: Principles, Methodology and Applications*; Academic Press: Oxford, 2014.

[2] Marchesini, S.; McGilvery, C. M.; Bailey, J.; Petit, C. Template-Free Synthesis of Highly Porous Boron Nitride: Insights into Pore Network Design and Impact on Gas Sorption. *ACS Nano* **2017**, *11*, 10003–10011.

[3] Xue, Y.; Dai, P.; Jiang, X.; Wang, X.; Zhang, C.; Tang, D.; Weng, Q.; Wang, X.; Pakdel, A.; Tang, C.; Bando, Y.; Golberg, D. Template-free synthesis of boron nitride foam-like porous monoliths and their high-end applications in water purification. *J. Mater. Chem. A* **2016**, *4*, 1469–1478.

[4] Tian, T.; Hou, J.; Ansari, H.; Xiong, Y.; L'Hermitte, A.; Danaci, D.; Pini, R.; Petit, C. Mechanically stable structured porous boron nitride with high volumetric adsorption capacity. *J. Mater. Chem. A* **2021**, *9*, 13366–13373.

[5] Bard, J. F. *Nonlinear parameter estimation*; Academic Press: NY, 1974; pp 61–70.

[6] Bötschi, S.; Rajagopalan, A. K.; Morari, M.; Mazzotti, M. Feedback Control for the Size and Shape Evolution of Needle-like Crystals in Suspension. IV. Modeling and Control of Dissolution. *Cryst. Growth Des.* **2019**, *19*, 4029–4043.

[7] Bouhlel, M. A.; Hwang, J. T.; Bartoli, N.; Lafage, R.; Morlier, J.; Martins, J. R. R. A. A Python surrogate modeling framework with derivatives. *Adv. Eng. Softw.* **2019**, 102662.

[8] Rajendran, A.; Kariwala, V.; Farooq, S. Correction procedures for extra-column effects in dynamic column breakthrough experiments. *Chem. Eng. Sci.* **2008**, *63*, 2696–2706.

[9] Joss, L.; Mazzotti, M. Modeling the extra-column volume in a small column setup for bulk gas adsorption. *Adsorption* **2012**, *18*, 381–393.

[10] Friedrich, D.; Mangano, E.; Brandani, S. Automatic estimation of kinetic and isotherm parameters from ZLC experiments. *Chem. Eng. Sci.* **2015**, *126*, 616–624.

[11] Wilkins, N. S.; Rajendran, A.; Farooq, S. Dynamic column breakthrough experiments for measurement of adsorption equilibrium and kinetics. *Adsorption* **2021**, *27*, 397–422.

[12] Levenspiel, O. *Chemical Reaction Engineering*, 3rd ed.; Wiley: New York, 1999; pp 281–287.

[13] Brandani, S.; Mangano, E. The zero length column technique to measure adsorption equilibrium and kinetics: lessons learnt from 30 years of experience. *Adsorption* **2021**, *27*, 319–351.
